# DNA damage triggers squamous metaplasia in human lung and mammary cells via mitotic checkpoints

**DOI:** 10.1038/s41420-023-01330-3

**Published:** 2023-01-21

**Authors:** Lucía San Juan, Ana Freije, Natalia Sanz-Gómez, Beatriz Jiménez-Matías, Cayetano Pleguezuelos-Manzano, J. Ramón Sanz, Ernesto de Diego, Sara Naranjo, Hans Clevers, Alberto Gandarillas

**Affiliations:** 1 Cell Cycle, Stem Cell Fate and Cancer Laboratory, Institute for Research Marqués de Valdecilla (IDIVAL), 39011 Santander, Spain; 2 Oncode Institute, Hubrecht Institute, Royal Netherlands Academy of Arts and Sciences and University Medical Center, Utrecht, The Netherlands; 3 Plastic Surgery Service, Hospital Universitario Marqués de Valdecilla, Santander, Spain; 4 Faculty of Medicine, Universidad de Cantabria, Santander, Spain; 5 Pediatric Surgery Service, Hospital Universitario Marqués de Valdecilla, Santander, Spain; 6 Service of Thoracic Surgery, Hospital Universitario Marqués de Valdecilla, Santander, Spain; 7 INSERM, Languedoc-Roussillon, 34394 Montpellier, France; 8 Present Address: Pharma, Research and Early Development (pRED) of F, Hoffmann-La Roche Ltd, Basel, Switzerland

**Keywords:** Differentiation, Cancer

## Abstract

Epithelial transdifferentiation is frequent in tissue hyperplasia and contributes to disease in various degrees. Squamous metaplasia (SQM) precedes epidermoid lung cancer, an aggressive and frequent malignancy, but it is rare in the epithelium of the mammary gland. The mechanisms leading to SQM in the lung have been very poorly investigated. We have studied this issue on human freshly isolated cells and organoids. Here we show that human lung or mammary cells strikingly undergo SQM with polyploidisation when they are exposed to genotoxic or mitotic drugs, such as Doxorubicin or the cigarette carcinogen DMBA, Nocodazole, Taxol or inhibitors of Aurora-B kinase or Polo-like kinase. To note, the epidermoid response was attenuated when DNA repair was enhanced by Enoxacin or when mitotic checkpoints where abrogated by inhibition of Chk1 and Chk2. The results show that DNA damage has the potential to drive SQM via mitotic checkpoints, thus providing novel molecular candidate targets to tackle lung SCC. Our findings might also explain why SCC is frequent in the lung, but not in the mammary gland and why chemotherapy often causes complicating skin toxicity.

## Introduction

Lung cancer is the most deadly malignancy causing about 2 million deaths per year worldwide (25% of cancer deaths) [1]. Squamous cancer or squamous cell carcinoma (SCC) is the second most common neoplasm, generally aggressive and one of the leading cause of death by cancer [2–4]. SCC typically appears in stratified epithelia of the skin, oral cavity, pharynx, larynx, oesophagus, or cervix [5], but is also common in the simple non-stratified epithelia of the lung [6]. SCC constitutes about 20% of cancer in the skin, 90% in head and neck, 20–80% in oesophagus, or 25–30% in lung [7]. Globally, SCCs cause 14% of cancer deaths (2.5% of overall deaths).

Lung SCC (LSCC) originates in the pseudostratified epithelium of the bronchi or the simple epithelium of the alveoli. It is often difficult to find SCC clinical specific studies within lung cancer, or within non-small cell cancer. However, SCC is reported around 30% of all lung cancer, 40% in men and 25% in women [6, 8, 9]. About 18% of patients with LSCC reach 5 years survival [10]. Although immunotherapy has bettered the outcome of some of these tumours [11, 12], still the general prognosis of LSCC is poor and there is lack of specific biomarkers.

In contrast to the lung, SCC is very rare in the simple epithelium of the mammary gland, accounting for less than 0.1% of all breast cancer [13–17]. Due to the rarity of this malignancy, there is not sufficient literature for an appropriate management and prognosis of the disease. However, some studies suggest that it is an aggressive type of tumour [13] with chronic inflammation being a risk factor [14].

It has been proposed that SCCs of the lung might arise from complicating benign squamous metaplasia (SQM) [6, 13, 14]. Benign SQM is a pre-invasive lesion that involves the remodelling of a simple or pseudostratified epithelium towards a stratified epidermoid squamous epithelium comprising multiple layers of cells. The new structure resulting from SQM might be more resistant to injury [18]. Lung SQM (LSQM) is usually asymptomatic [6] and seems to be reversible after removal of the irritant, spontaneously or upon vitamin A that appears to preserve the secretory phenotype [19, 20]. LSQM often precedes LSCC and lung cells must undergo squamous differentiation during the genesis of SCC. Benign SQM is common in the lung, both in the airway or the alveolar epithelium and is associated with tissue irritation and dysplasia [18].

The molecular or cellular trigger of SQM is unknown, as it is unclear why it is so frequent in the lung and so rare in the mammary gland. Given the high frequency of SQM and the potential to progression into cancer, studying the mechanisms involved would provide new grounds for the prevention and therapy of aggressive LSCC.

We previously identified a novel response that induces squamous differentiation in epidermal cells after irreparable DNA damage [21]. We hypothesised that the cellular pathways responding to DNA damage (DDR) might be part of the squamous phenotype and drive SQM. To test this, we freshly isolated epithelial cells from the human lung or mammary gland and investigated their response to irreparable DNA damage and to mitotic checkpoints. Simply the proliferative stimulation causing replication stress drove lung and mammary cells into mild SQM. Lung and mammary cells rapidly underwent squamous differentiation in response to genotoxic agents or to sustained inhibition of mitosis. Lung organoids also expressed squamous markers in response to genomic damage. Interestingly, reinforcing DNA repair or inhibiting Chk1 and Chk2 G2/M checkpoint molecules attenuated the squamous response. Our results indicate that mitotic checkpoints in response to DNA damage drive pre-malignant SQM. This might explain why squamous cancer is so frequent in the lung, especially in association with smoking, whereas it is so rare in epithelia that are less exposed to carcinogens, such as the mammary gland. The findings provide new insight for the detection and therapy of LSCC.

## Results

**Fig. 1:**
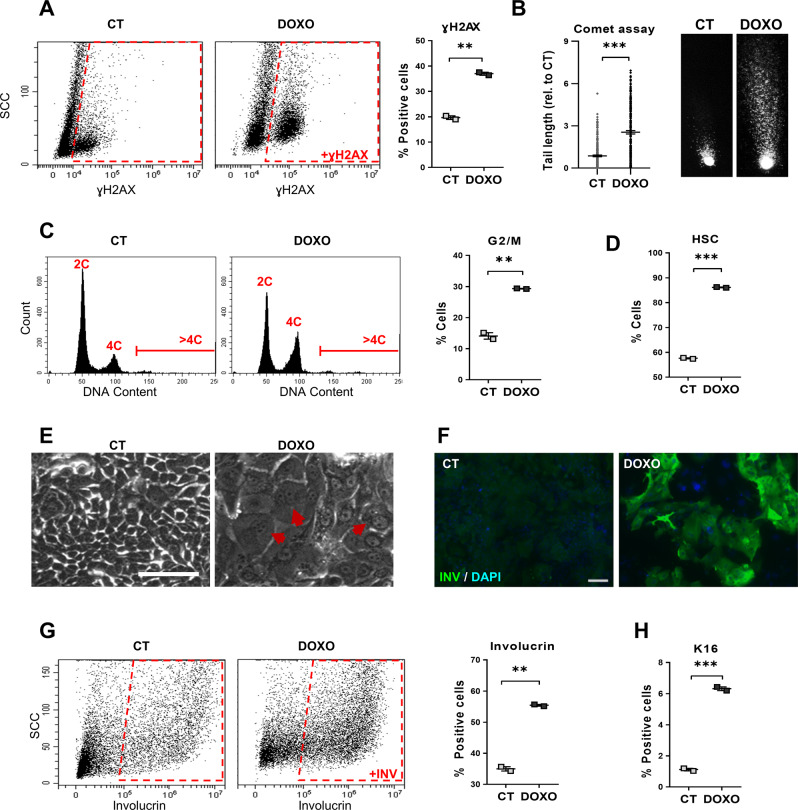
Doxorubicin induces squamous metaplasia in human lung epithelial cells. Human primary lung epithelial cells were treated with the dimethyl sulfoxide vehicle (CT) or with 0.5 μM Doxorubicin (DOXO) for 24 h (**A**, **C**) or 48 h (**B**, **D**–**H**). **A** Representative flow-cytometry analysis for the DNA damage marker γH2AX (+γH2AX, positive cells). Quantitation in the right histogram. **B** DNA fragmentation as analysed by comet assays, measured by tail length relative to CT (*n* = 245–247). Photographs show representative images of nuclei in CT or DOXO-treated cells as indicated. **C** Representative flow-cytometry analyses of DNA content of cells (2C, 4C, and >4C indicate diploid, mitotic/tetraploid and polyploid cells, respectively). Plot on the right shows percent of CT or DOXO cells in the G2/M phase of the cell cycle. **D** Percent of CT or DOXO-treated cells with high light scatter (HSC) typical of squamous differentiation, as analysed by flow cytometry. **E** Representative phase contrast images of cells treated for 48 h as indicated. Red arrows point at polyploid cells with several or large nuclei. Scale bar, 50 μm. **F** Immunofluorescence for the squamous differentiation marker involucrin (green). Blue is nuclear DNA by DAPI. Scale bar, 100 μm. **G** Representative flow cytometry analysis for involucrin (+INV, positive cells). The percent of involucrin positive CT or DOXO-treated cells is shown on the right. **H** Percent of squamous marker keratin K16 positive cells, as analysed by flow cytometry. Flow cytometry analysis gates were stablished according to negative isotype antibody control. Data are mean ± SEM of duplicate samples, representative of 2–3 independent experiment from two different human individuals with similar results. ***p ≤ 0.001, **p ≤ 0.01.

We freshly isolated epithelial cells from human lung distal parenchyma. Hyperactivation of the cell cycle by growth factors or oncogenes causes replication stress [22–24]. The stimulatory culture conditions in the presence of serum and growth factors caused a certain level of DNA damage and chromosomal fragmentation, as measured by the early marker γH2AX and by comet assays (CT; Fig. 1A, B). We treated lung cells with Doxorubicin (DOXO), a genotoxic drug, in order to induce genetic damage. As expected, the DOXO treatment strongly induced both γH2AX and DNA fragmentation (DOXO; Fig. 1A, B). DNA damage triggers the G2 and mitosis (G2/M) checkpoints [25]. Consistently, DOXO-treated cells accumulated in G2/M with 4C DNA content (Fig. 1C). DOXO-induced DNA damage caused a morphological change of cells that is typical of epidermoid differentiation [26, 27], involving increased size and complexity, as monitored by flow cytometry (high scatter; HSC; Fig. 1D and Supplementary Fig. 1A) and phase contrast microscopy (Fig. 1E). We subsequently analysed markers of stratified squamous epithelia in the lung cultures. Involucrin is specific of all squamous epithelia including the epidermis, oral mucosa, head and neck or oesophagus [28]. Keratin K13 is typically expressed in stratified epithelia of mucosa [29] and keratin K16 is induced in stratified layers of squamous epithelia upon stress [30]. A proportion of control lung cells in the growth-stimulatory conditions expressed some squamous markers (Fig. 1F–G and Supplementary Fig. 1B; CT). However, the acute DNA damage caused by DOXO induced a striking rise of the proportion of cells expressing involucrin, K16 and K13 (Fig. 1F–H and Supplementary Fig. 1B, C). The sustained 48 h DOXO treatment eventually triggered squamous terminal differentiation, as it suppressed the capacity of cells to proliferate in the absence of the drug (Supplementary Fig. 1D). We detected no significant Sub-G1 cell population, due to early chromosomal fragmentation, typically found in apoptotic adherent cells [30], in any cell cycle analysis (Fig. 1C). The mere subculture of lung cells in the hyperproliferative culture conditions progressively induced an evident epidermoid phenotype (Supplementary Fig. 1E).

**Fig. 2:**
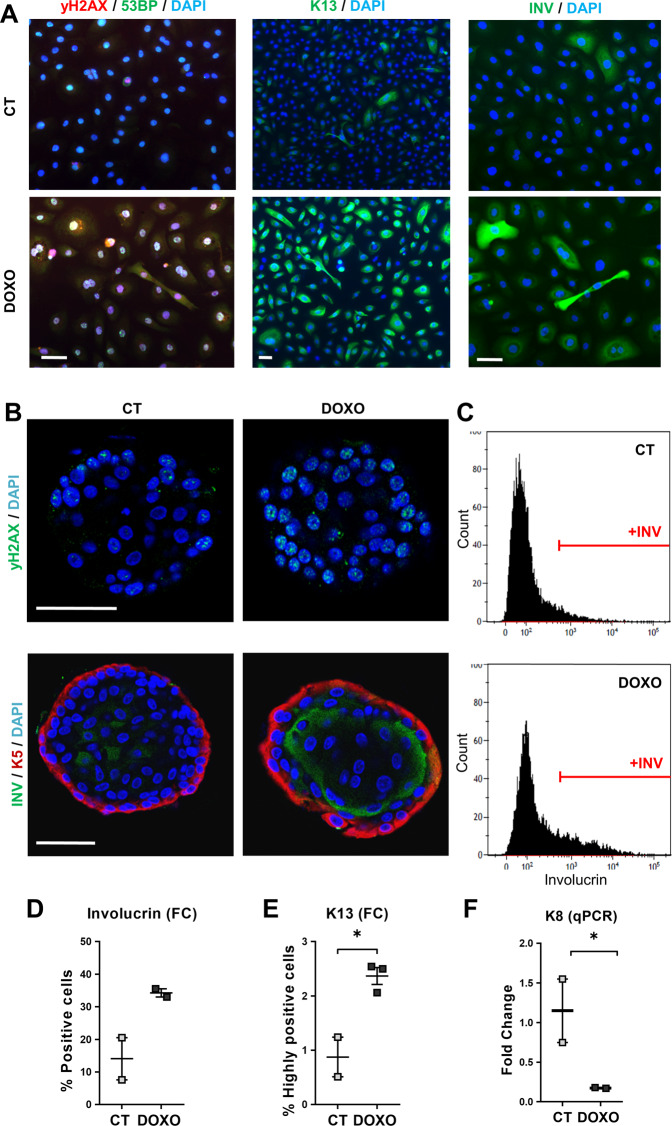
Doxorubicin induces squamous differentiation in human lung epithelial cells in a serum-free lung-adapted medium and in organoid reconstructions. **A** Human primary lung epithelial cells isolated and cultured in lung-adapted medium were treated for 48 h with the dimethyl sulfoxide vehicle (CT) or with 0.5 μM Doxorubicin (DOXO), as indicated. Immunofluorescence for 53BP (green) and γH2AX (red), for K13 (green), or for involucrin (green), as indicated. Blue is nuclear DNA by DAPI; scale bar, 50 μm. Representative of two independent experiments with similar results. **B**–**F** Airway lung organoids (AOs) in organoid expansion medium were treated with dimethyl sulfoxide (CT) or with 0.25 μM Doxorubicin (DOXO) for 24 h (**C**, **D**, **F**) or 48 h (**B**, **E**), as indicated. **B** Immunofluorescence confocal images of AOs treated as indicated and stained for γH2AX (top; green) or for involucrin (bottom; green) and K5 (red). Scale bar, 50 μm. Blue is nuclear DNA by DAPI. **C** Representative flow cytometry analyses for the differentiation marker involucrin (+INV, positive cells). **D** Percent of involucrin positive cells by flow cytometry. **E** Percent of keratin K13 positive cells by flow cytometry. **F** mRNA fold change of keratin K8 by qPCR. Positive cells by flow cytometry were gated according to negative isotype antibody control. Data are mean ± SEM of two or three replicate samples of two independent experiments from two different human individual. *p ≤ 0.05, *p* for +INV = 0.09.

Since the above Rheinwald culture conditions cause replication stress and DNA damage, especially due to the presence of serum and growth factors [21], we alternatively cultivated lung cells in a lung-adapted medium without serum. All lung cultured cells, in either medium, homogenously expressed keratin K5, marker of basal cells [31] (Supplementary Fig. 2A). As shown in Supplementary Fig. 2B, untreated cells in the adapted serum-free medium expressed scarce squamous markers and did not stratify, in contrast to cells in the Rheinwald medium. However, treatment with DOXO in the same conditions strikingly induced DNA damage and all tested squamous markers (Fig. 2A and Supplementary Fig. 2C–G). To further investigate the capacity of lung cells to undergo SQM in response to DNA damage, we tested an organoid, more physiologically mimicking organotypic culture [32]. We generated lung organoids from human patients. As shown in Fig. 2B, a 48 h DOXO treatment induced a significant rise of DNA damage that we quantitated by the expression of γH2AX. In addition, we detected an induction of involucrin or K13 squamous suprabasal proteins and a decrease of lung simple epithelial marker keratin K8 (Fig. 2B–F).

**Fig. 3:**
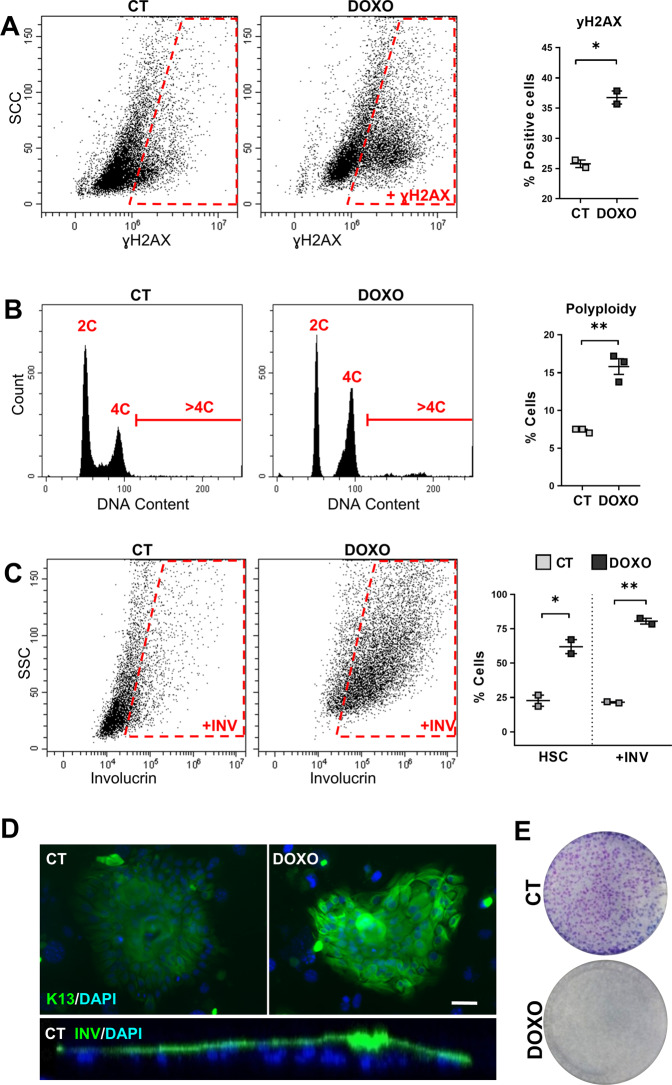
Doxorubicin induces squamous metaplasia in human mammary gland epithelial cells. Human primary mammary gland epithelial cells were treated with dimethyl sulfoxide (CT) or with 0.5 μM Doxorubicin (DOXO) for 48 h, as indicated. **A** Representative flow cytometry analysis for the DNA damage marker γH2AX (+γH2AX, positive cells). The percent of +γH2AX is shown on the right. **B** Representative flow cytometry analyses of DNA content (2C, 4C, and >4C). The percent of polyploid cells (>4C) is shown on the right. **C** Representative flow-cytometry analysis for the differentiation marker involucrin (+INV, positive cells). The percent of cells with high light scatter (HSC), or +INV is shown on the right, as indicated. **D** Top: representative mammary gland colonies after immunofluorescence for keratin K13 (green), treated as indicated. Blue is nuclear DNA by DAPI. Scale bar, 50 μm. Bottom: Orthogonal view profile of a CT mammary gland colony after immunofluorescence for involucrin (INV). **E** Clonogenic capacity of cells plated after 48 h treatment as indicated and drug-released. Positive cells by flow cytometry were gated according to negative isotype antibody control. Data are mean ± SEM of duplicate or triplicate samples, representative of two independent experiment from two different human individuals with similar results. **p ≤ 0.01, *p ≤ 0.05.

The results altogether show that DOXO-induced DNA damage triggers SQM in lung cells. We questioned whether this response might be common to other non-squamous epithelia. We chose the simple epithelium of the mammary gland where, in contrast to the lung, SQM or SCC is very infrequent. We isolated epithelial cells from human healthy mammary gland (from plastic surgery) and subjected them to DNA damage upon DOXO. As expected, DOXO treatment caused induction of γH2AX and accumulation of cells in G2/M (Fig. 3A, B). In addition, a proportion of mammary cells during the time of the treatment underwent polyploidisation (Fig. 3B). Interestingly, untreated control cells in the presence of serum and growth factors resembled epidermal keratinocytes (Supplementary Fig. 3A) and homogenously expressed keratin K5 (Supplementary Fig. 3B), typical of mammary basal myoepithelial cells. Although various cell subtypes expressing or not keratin K5 populate the epithelium of the lung or the mammary gland, this suggests that we selected basal K5 positive cells because they retain the amplifying proliferative capacity of the epithelium. In these conditions untreated cells, although at a minor extent, expressed squamous markers and displayed a high capacity for stratification (Fig. 3C, D, Video 1 and Supplementary Fig. 3C, D, CT). As observed for lung cells, DOXO induced in mammary cells a striking expression of squamous markers involucrin and keratins K13 and K16, as well as morphological changes typical of squamous differentiation (Fig. 3C, D and Supplementary Fig. 3C–E). As a result of DOXO treatment, mammary cells ultimately lost the capacity to proliferate, indicative of squamous differentiation (Fig. 3E). No sign of early apoptotic sub-G1 cells due to early DNA fragmentation was observed in any of the cell cycle analyses (Fig. 3B).

**Fig. 4:**
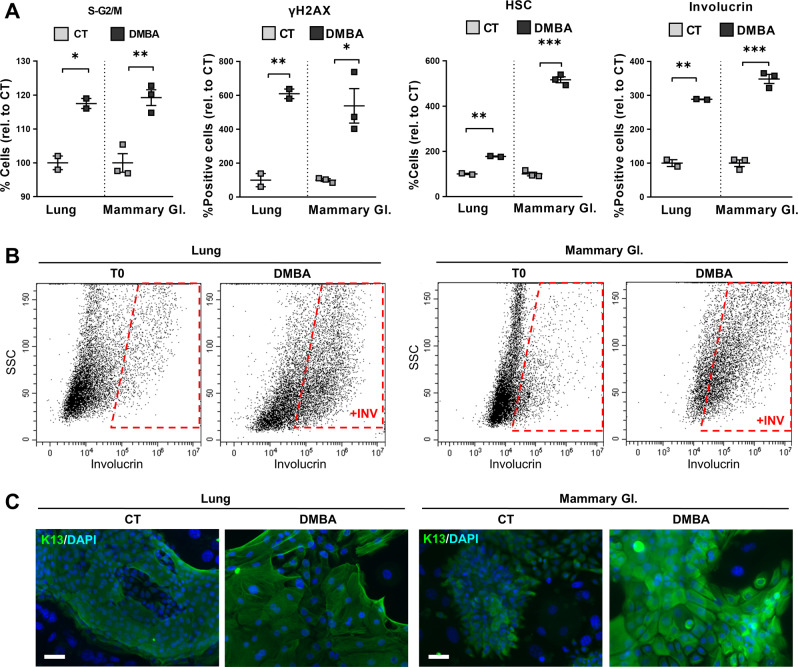
Lung and mammary gland epithelial cells undergo squamous metaplasia upon tobacco carcinogen DMBA. Human primary lung or mammary gland (Mammary Gl.) epithelial cells were treated with dimethyl sulfoxide (CT) or with 1 μg/ml DMBA for 24 h (A, S-G2/M, and yH2AX) or 72 h (**A**, HSC and involucrin, **B**, **C**). **A** Flow cytometry quantitations for the percent of, from left to right: cells in the S-G2/M phase of the cell cycle, γH2AX positive cells, cells with high light scatter (HSC) typical of squamous differentiation, involucrin positive cells, all relative to CT. **B** Representative flow cytometry analyses for the differentiation marker involucrin. +INV, positive cells; T0 = untreated cells. **C** Representative immunofluorescence for keratin K13 (green) upon the indicated treatments. Blue is nuclear DNA by DAPI. Scale bar, 50 μm. Positive cells by flow cytometry were gated according to negative isotype antibody control. Data are mean ± SEM of two or three replicate samples, representative of 2–3 independent experiment from two different human individuals with similar results. ***p ≤ 0.001, **p ≤ 0.01, *p ≤ 0.05.

We concluded that lung and mammary cells have a similar intrinsic capacity to undergo SQM in response to DNA damage. SQM in the lung is highly associated with cigarette smoking and one of the main genotoxic substances in the smoke is the carcinogen 7,12-Dimethylbenz(a)anthracene (DMBA) [33]. Therefore, we treated lung or mammary gland epithelial cells with DMBA for 72 h and investigated their capacity to undergo squamous differentiation. We confirmed that DMBA caused cell cycle arrest in S/G2/M phases and DNA damage, by DNA content analyses and the expression of γH2AX (Fig. 4A, and Supplementary Fig. 4A, B). DMBA also caused a striking rise of large cells with squamous morphology and expression of involucrin and K13 squamous markers (Fig. 4A–C and Supplementary Fig. 4C). As a result of the G2/M block and the resulting squamous differentiation, most cells lost their proliferative capacity after drug release (Supplementary Fig. 4D).

**Fig. 5:**
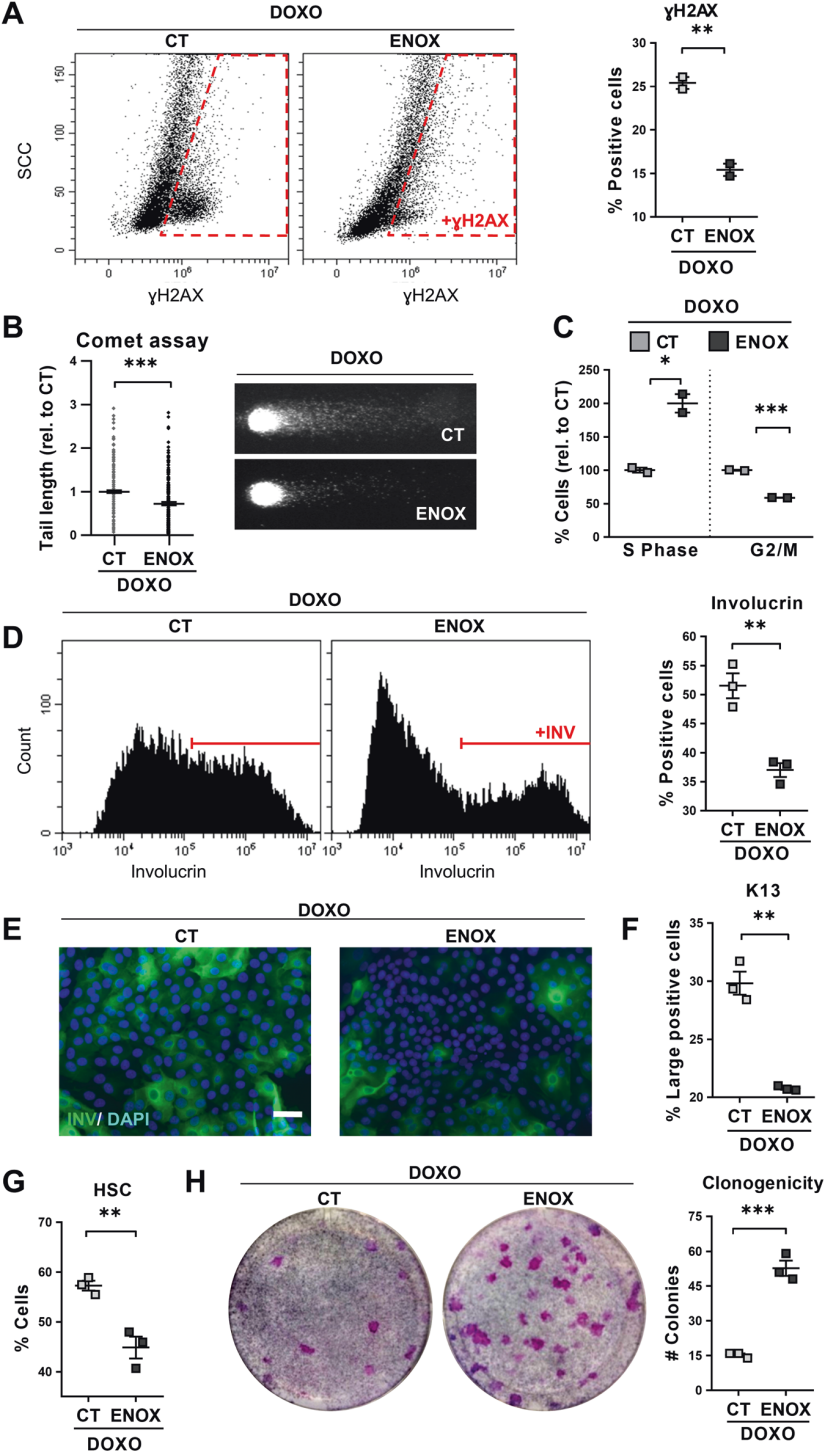
Enhancement of DNA repair by Enoxacin suppresses Doxorubicin-induced squamous metaplasia in lung epithelial cells. Human primary lung epithelial cells were treated with Doxorubicin (DOXO) and with the dimethyl sulfoxide vehicle (CT) or with 200 μM Enoxacin (ENOX). **A** Representative flow-cytometry analyses for the DNA damage marker γH2AX of cells treated for 24 h, as indicated (γH2AX+, positive cells). Scattered plot (right) shows percent of γH2AX positive cells. **B** DNA fragmentation as analysed by *comet* assays after 24 h treatment as indicated and measured by tail length relative to CT (*n* = 237–312). Photographs show representative images of nuclei in CT or ENOX-treated cells, as indicated. **C** Percent of cells in the S or G2/M phases of the cell cycle relative to CT, analysed by flow cytometry after 24 h with the indicated treatments. **D** Representative flow cytometry analyses for involucrin (+INV, positive cells) after 48 h with the indicated treatments. Positive cells were gated according to negative isotype antibody control. The percent of involucrin positive CT or ENOX-treated cells is shown on the right. **E** Immunofluorescence for involucrin (green) of cells treated for 48 h, as indicated. Blue is nuclear DNA by DAPI. Scale bar, 50 μm. **F** Percent of large keratin K13 positive cells analysed by flow cytometry after 24 h. **G** Percent of cells with high light scatter (HSC), analysed by flow cytometry after 24 h. **H** Clonogenic capacity of cells drug-released and plated after 48 h with the indicated treatments. The number of CT or ENOX colonies is shown on the right plot. Positive cells by flow cytometry were gated according to negative isotype antibody control. Data are mean ± SEM of 2–3 replicate samples, representative of 2–3 independent experiment from two different human individuals with similar results. ***p ≤ 0.001, **p ≤ 0.01, *p ≤ 0.05.

We aimed to elucidate whether unrepaired DNA damage was responsible for DOXO-induced squamous differentiation. Enoxacin is an antibiotic molecule that has been shown to act as small-molecule enhancer of microRNA (SMER) [34]. Recently, it has been described that Enoxacin boosts DNA repair in human cells via non-coding RNAs [35]. Addition of Enoxacin to lung cells attenuated the expression of γH2AX and the presence of DNA fragments caused by DOXO (Fig. 5A, B and Supplementary Fig. 5A). Consistently, Enoxacin diminished the proportion of cells arrested in G2/M and increased the proportion of cells in the S phase of the cell cycle (Fig. 5C and Supplementary Fig. 5B). To note, Enoxacin also attenuated the squamous differentiation response to DOXO, as measured by expression of involucrin, keratin K13, or cell size and morphology (Fig. 5D–G and Supplementary Fig. 5C). Interestingly, Enoxacin also bettered the clonogenic capacity after sustained 48 h DOXO treatment (Fig. 5H).

**Fig. 6:**
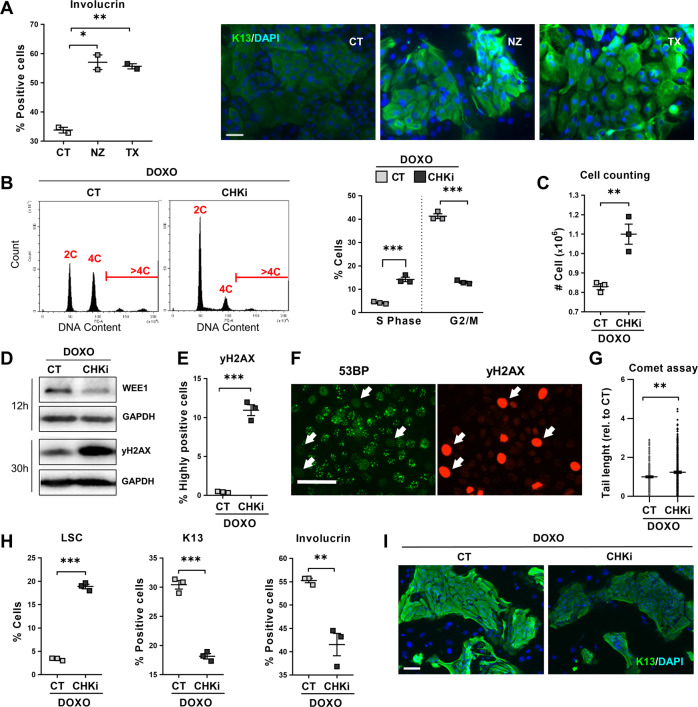
The G2/M checkpoints drive squamous metaplasia in response to DNA damage. **A** Human primary lung epithelial cells were treated with the dimethyl sulfoxide vehicle (CT), 20 μM Nocodazole (NZ, microtubule polimerisation inhibitor) or 200 μM Paclitaxel (TX, microtubule depolimerisation inhibitor). Left: Percent of CT, NZ, or TX involucrin positive cells, analysed by flow cytometry. Right: Immunofluorescence for involucrin (green). Blue is nuclear DNA by DAPI. Scale bar, 50 μm. **B**–**I** Human primary lung epithelial cells were treated with 0.07 μM Doxorubicin (DOXO) and with the dimethyl sulfoxide vehicle (CT) or with 0.5 μM AZD7762 (CHKi, Chk1/Chk2 inhibitor). **B** Representative flow-cytometry analyses of DNA content after the 24 h treatments as indicated. Plot on the right shows percent of CT or CHKi-treated cells in the S or G2/M phase of the cell cycle, as indicated. **C** Cell counting of recovered CT and CHKi-treated cells after 48 h treatment. **D** DNA damage marker γH2AX and Wee1 by Western blotting in cells treated for 12 or 30 h, as indicated. Glyceraldehyde 3-phosphate dehydrogenase (GAPDH) as loading control. **E** Percent of highly γH2AX positive cells, analysed by flow cytometry after 24 h. **F** Double immunofluorescence for 53BP (green) and γH2AX (red) in CHKi-treated cells at 24 h. Scale bar, 50 μm. White arrows point at strongly positive γH2AX cells lacking 53BP foci (typical of active DNA repair). **G** DNA fragmentation as analysed by comet assays after 24 h with the indicated treatments, measured by tail length relative to CT (*n* = 196–298). **H** Percent of low light scatter (LSC, left) cells, keratin K13 positive cells (centre) or involucrin positive cells (right), as analysed by flow cytometry (K13, 24 h-treatment; LSC and involucrin, 48 h-treatment). **I** Immunofluorescence of CT and CHKi-treated cells for keratin K13 (green) after 24 h treatments. Blue is nuclear DNA by DAPI. Scale bar, 100 μm. Positive cells by flow cytometry were gated according to negative isotype antibody control. Data are mean ± SEM of duplicate or triplicate samples, representative of 2–3 independent experiment from two different human individuals with similar results. ***p ≤ 0.001, **p ≤ 0.01, *p ≤ 0.05.

The DDR pathways induce the mitotic checkpoints that halt cells at G2 or mitosis [25]. To test whether DNA damage induces SQM in lung or mammary cells via the mitotic checkpoints, we directly blocked mitosis in lung or mammary gland cells. We made used of Nocodazole (NZ) that inhibits the polymerisation of microtubule and mitosis entry from G2, or Paclitaxel (Taxol; TX) that inhibits microtubuli depolymerisation and mitosis exit from metaphase. These drugs are used in chemotherapy. As shown in Supplementary Fig. 6A–D, either drug induced an accumulation of cells in G2/M and a typical squamous differentiation change in cell size and morphology both in lung and in mammary cells. Consistently, NZ or TX also strikingly induced the expression of squamous markers as measured by flow cytometry and immunofluorescence (Fig. 6A and Supplementary Fig. 7A–D). We observed a similar squamous response upon molecular inhibitors of the essential mitotic proteins AurBK or PLK1 (Aur-i or Plk-i; Supplementary Fig. 7B). As for Doxorubicin treatment, the mitosis block also resulted in a significant increase in the proportion of polyploid cells (Supplementary Fig. 6A, B) and eventual loss of clonogenic capacity, associated with late squamous differentiation (Supplementary Fig. 7E, F). As before, we found no apoptotic Sub-G1 cells due to early DNA fragmentation in the cell cycle analyses, apart from a minimal proportion in mammary cells upon NZ (<3%; Supplementary Fig. 6B).

To further elucidate whether the mitotic checkpoints control the epithelial phenotypic choice, we made use of a molecular inhibitor of essential checkpoint proteins Chk1 and Chk2 (CHKi) [25]. We then compared the response of lung cells to DOXO in the absence (CT) or presence (CHKi) of the inhibitor. The G2/M checkpoints halt cells in G2 and delay entry in mitosis to allow DNA repair. Consistently, abrogation of the G2/M checkpoints by CHKi remarkably suppressed the accumulation of cells in these phases of the cell cycle allowing cells to return into G1 and S phases (4C DNA content; Fig. 6B). Consequently, a higher number of cells was recovered in the presence of CHKi (Fig. 6C). This was a result of a faster and more efficient completion of mitosis, what was confirmed by the lack of accumulation of G2 cyclin A, cytoplasmic cyclin B or mitotic cdk1 (Supplementary Fig. 8A, B). Concomitantly, there was a significant rise of cells undergoing metaphase (pH3; Supplementary Fig. 8A) and a drop in the expression of mitosis inhibitor wee1 (Fig. 6D). Wee1 is a key component of the mitotic checkpoints. These results show that the G2/M checkpoint was alleviated by CHKi and cells were allowed to divide. However, these cells likely due to impaired DNA repair in G2, beared a high index of DNA damage as monitored by γH2AX (Fig. 6D, E and Supplementary Fig. 8C). Interestingly, strongly positive γH2AX cells lacked the DNA repair marker 53BP protein (Fig. 6F and Supplementary Fig. 8D). Physical evidence of a reduction in DNA repair was the higher level of DNA fragmentation in the presence of the checkpoint inhibitor (Fig. 6G and Supplementary Fig. 8E).

To determine whether the G2/M checkpoints were responsible of SQM upon DNA damage, we evaluated squamous markers in the presence of DOXO and the CHKi. As shown in Fig. 6H–I and in Supplementary Fig. 8F–H, the abrogation of the G2/M checkpoints attenuated the squamous morphological changes and the induction of K13 and involucrin squamous proteins.

## Discussion

Even though LSCC is a major cause of death worldwide, the mechanisms driving squamous cancer in non-squamous epithelia remain largely unexplored. Our results identify a cellular pathway with the potential to control the epithelial phenotype and to induce SQM. Altogether the results show that SQM, a first step to squamous cancer, responds to genotoxic stress.

SQM is typically associated with tissue irritation, injury, oxidative stress or chronic inflammation in the non-squamous epithelia of the bladder, endocervix or the lung or very rarely, in the mammary gland [6, 18, 36, 37]. However, the cause-effect relationship of this transdifferentiation phenomenon has not been established. In the mammary gland, SCC is also very rare, further suggesting that SQM is a premalignant prerequisite. The main question underlying this issue is whether the capacity to undergo SQM is intrinsic to cells within a tissue (niche) or it depends on environmental cues. By isolating the epithelial cells and placing them in the same conditions, we could study their behaviour independently of the biological niche. The finding that proliferative lung and mammary gland cells in vitro undergo SQM in response to DNA damage caused by genotoxic agents, such as the carcinogen DMBA, suggests that it is the environmental factors rather than intrinsic properties of the cells or the niche what controls epithelial plasticity. This might explain why LSCC is strongly associated with smoking. The mammary gland is far less exposed to genotoxic hazard than the lung.

According to our results, the endogenous pathways responding to DNA damage (DDR) would be involved in defining the squamous phenotype. It is tempting to speculate that genotoxic damage itself developed the stratified epithelia as a reaction to genetic insult in the skin or head and neck. Epithelia that are less exposed to genotoxicity such as the mammary gland, or the lung in the absence of acute pollutants, would maintain the non-squamous phenotype. As squamous differentiation seems to protect cells from apoptosis [38, 39], it might be the conservative alternative fate in non-squamous epithelia upon unrepaired genetic damage [38, 39], in order to constitute a stronger barrier to damage and injury. This is consistent with lung and mammary cells expressing epidermoid markers in a replication stress-inducing medium.

The squamous differentiation response of lung cells appears to be induced specifically by the DDR, as it was attenuated when DNA repair was enhanced by Enoxacin. However, DNA damage induces the mitotic checkpoints, which in turn inhibit progression of mitosis [25]. The fact that a mere block of mitosis by physical o biochemical means triggered squamous differentiation in lung or mammary gland cells, suggests that mitotic checkpoints drive the epidermoid phenotype in non-stratified epithelial cells. This model was further supported when we suppressed the DOXO G2/M arrest in lung cells by use of the inhibitor of the key checkpoint proteins Chk1,2. This inhibited epidermoid differentiation in spite of a high degree of DNA damage, suggesting that the G2/M are required for SQM.

Keratinocytes have the capacity to undergo mitotic slippage and to become polyploid upon a sustained G2/M arrest [40, 41]. The block of G2/M in lung or mammary gland cells induced a proportion of polyploid cells. Polyploidisation is a mechanism that various human tissues utilise when large cells and a high production of protein is required [42]. Interestingly, mammary cells were recently shown to become polyploid during lactation [43]. During the great growth that takes place at the branching phase of the gland prior to lactation, mammary cells are driven into rapid and very active proliferation. It is tempting to speculate that those cells undergo replication stress-induced mitotic block and polyploidy. Within these lines, proliferative rates in the adult steady-state lung are low, cells generally being quiescent, whereas SQM is associated with hyperproliferation [31, 44].

**Fig. 7:**
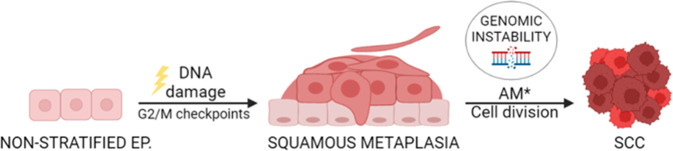
Model for the role of DNA-damage and mitotic checkpoints in the development of squamous metaplasia and subsequent squamous cancer in non-stratified epithelia. Chronic or acute exposure of non-squamous epithelia to genotoxic agents would drive G2/M arrest via the DNA damage response. In turn, G2/M checkpoints would drive benign hyperplastic squamous differentiation (squamous metaplasia). Unrepaired cells would undergo terminal post-mitotic squamous differentiation. In the event of additional mutations (AM) hitting the G2/M checkpoints, cells overcoming the cell division block would proliferate bearing genomic instability, eventually resulting in cancer. Ep: epithelium. Created with BioRender.com.

The results have implications into cancer and the toxicity that many cancer chemotherapeutic treatments cause. Alterations in the mitotic checkpoints during benign SQM in lung cells chronically exposed to genotoxic insult might eventually allow them to bypass the mitotic block. This would promote proliferation with a high degree of genomic instability (Fig. 7) thus promoting cancer progression. These cells might be at the origin of aggressive SCC. On the other hand, chemotherapeutics targeting mitosis might induce SQM in the healthy epithelia as part of the toxic effects. For instance, eccrine SQM is frequently part of the skin toxicity upon chemotherapy, also referred to as toxic erythema of chemotherapy [45–49]. Skin toxicity is sometimes so acute that the anticancer treatment must be interrupted. The mechanisms driving toxic erythema of chemotherapy remain to be elucidated.

In summary, our results show that human epithelial cells retain a great plasticity and point at a cellular pathway leading to SQM in simple epithelial cells. The findings provide new grounds for understanding and preventing the usually aggressive squamous cancer in non-squamous organs. In addition, they might reveal a mechanism for the frequent and complicating skin eccrine toxicity caused by chemotherapy.

## Materials and methods

### Ethical

Ethical permission for this study was requested, approved, and obtained from the Ethical Committee for Clinical Research of Cantabria Council, PI20/00880 (Spain). Human tissue material discarded after surgery was obtained with written consent presented to the patients, and it was treated anonymously. Airway organoid lines used in the study were under the protocol Z-12.55 approved by the “Verenigde Commissies Mensgebonden Onderzoek” of the St. Antonius Hospital Nieuwegein (The Netherlands).

### Cell culture and chemical treatments

Biopsies were included from 6 human individuals, 4 from distal lung epithelium and 2 from mammary gland epithelium. Three lung patients were paediatric (non-smokers). One lung patient was an adult smoker (59). No significant differences were observed in the original preparation of cells. Very similar results were obtained in all assays among individuals.

Primary epithelial cells were isolated from non-lesional distal lung parenchyma. Biopsies were washed in Wash buffer: 100 U/mL penicillin-streptomycin (Pen/Strep; BE17-602E, Lonza) and 0.5 µg/mL Amphotericin B (11510496, Gibco), 1× PBS (BE17-517Q, Lonza) and cut into small pieces (5 mm Ø approximately). Pieces were again abundantly washed with Wash buffer. Pieces were then incubated with 0.25% trypsin (25200-056, Thermo-Fisher)-1 mM EDTA for 1 h at 37 °C under constant rotation. After incubation, the supernatant was discarded and the remaining pieces were incubated in Digestion solution: 0.075% trypsin, 2.5 mg/mL collagenase P (11213865001, Roche) in Wash buffer, for 45 min at 37 °C under rotation. Trypsin was then inactivated by the addition of 10% serum Rheinwald-flavin adenine dinucleotide medium (FAD medium) [50]. Isolated cells were counted and plated at a density 5,300–17,600 cell/cm^2^ in FAD medium (1ug/ml EGF, 5% fetal calf serum and 1.2 mM Ca^+2^) supplemented with Penicillin-Streptomycin 100 U/ml (P/S. Lonza; ref. BE17–602E), in the presence of a mouse J2-3T3 fibroblast feeder layer (inactivated by mitomycin C), as described [50]. Remaining pieces were subjected to another round of trypsin digestion, counted, and plated as before.

For isolating of mammary gland epithelial cells, non-lesional mammary biopsies were processed as for lung, except no 0.25% trypsin-1 mM EDTA incubation was performed and pieces were digested for 30 min with the Digestion solution up to 4 times.

Low culture passages 1 to 4 were used in all cases. J2-3T3 mouse fibroblasts were cultured in Dulbecco medium 10% bovine calf serum (35-053-CM, Corning), 2 mM L-glutamine (17605E, Lonza), and supplemented with pen-strep 100 U/ml.

For primary lung epithelial cells, specific Airway Epithelial Cell Growth Medium from Promocell (C-21060) supplemented with pen-strep 100 U/ml was also used (‘lung-adapted medium’). Cells here were isolated as described before and plated in this medium, at 59,000–99,000 cells/cm^2^. Low culture passages 1 to 4 were used.

For 3D lung cultures, human airway organoids (AOs) were prepared from bronchoalveolar lavages of two different healthy human individuals and cultured in AO medium as described by Sachs et al. [32]. Briefly, lung cell pellets were resuspended in cold Cultrex growth factor reduced Basement Membrane Extract (BME, Trevigen‐3533‐010‐02) and plated in drops. Upon complete gelation of BME at 37 °C, AO medium was added and AOs were cultured at 37 °C/5% CO_2_. Single‐cell suspensions were initially seeded at high density and reseeded at a lower density after 1 week as described. These organoids grow as spheres. Organoids were treated for 24 or 48 h with Doxorubicin and then harvested for quantitative PCR, immunofluorescence, or flow cytometry. In spite of certain variability in the response, we observed an induction of epidermoid markers in both strains. For one of the strains, the induction of the epidermoid phenotype was confirmed by the expression of the epidermoid marker involucrin (Fig. 2B–D). In the second individual, the epidermoid conversion was assessed by the induction of K13 and the inhibition of K8 (Fig. 2E, F). Given the tendency of the results and our experience, we found the variability of the expression of epidermoid markers between the two organoid strains likely due to variations in the timing of the response.

Cultures were routinely tested and proved free of mycoplasma contamination.

Cultures were treated, for the lengths of time indicated throughout, with the following compounds dissolved in the corresponding medium: 20 μM Nocodazole (M1404; Sigma-Aldrich, St Louis, MO); 100 nM Taxol (Paclitaxel T7402, Sigma-Aldrich); 0.04, 0.07 or 0.5 mM Doxorubicin (DOXO; 44583; Sigma-Aldrich); 2 μM of the AurBK inhibitor (ZM447439; Tocris Bioscience, Bristol, UK); 100 nM of the PLK1 inhibitor (BI2536; Axon MedChem BV, Netherlands); 1 μg/mL 7,12-DMBA (D-3254; Sigma-Aldrich); 200 μM Enoxacin (S1756, Selleckchem); 0.5 μM the Chk1, 2 inhibitor (AZD7762; C-8052, Sigma-Aldrich). All drugs were dissolved in dimethyl sulfoxide (DMSO). Parallel cultures were always subjected to the DMSO vehicle as control samples.

### Clonogenicity assay

For clonogenicity assays, epithelial adherent cells were plated at very low density (5,000 cells per T6 well in triplicate samples: 500 cell/cm^2^) in FAD medium. About 9 to 16 days later, the cultures were stained with rhodanile blue, as described previously [51].

### Antibodies

The following primary antibodies were used: mouse anti-p-H2AX Ser139 (sc-517348, Santa Cruz Biotechnology; JBW301; immunofluorescence, IF, and flow cytometry FC), rabbit anti-53BP (A300-272A, Bethyl Laboratories; IF), mouse anti-involucrin (SY3; lab made, Hudson et al., 1992; IF and FC), mouse anti-K13 (Novus Biologicals, Littleton, CO; FC and IF), mouse anti-K16 (sc-53255, Santa Cruz Biotechnology; IF and FC), mouse anti-p21CIP (CP74, Sigma-Aldrich; WB), mouse anti-glyceraldehyde 3-phosphate dehydrogenase (GAPDH; sc-47724; Santa Cruz Biotechnology; WB), mouse anti-α-tubulin (B-5-1-2, Santa Cruz Biotechnology, sc-23948), mouse anti-wee1 (B11, Santa Cruz Biotechnology, sc-5285, WB), rabbit anti-Cyclin-A (H432, Santa Cruz Biotechnology, sc-751; WB and IF), mouse anti-Cyclin-B (Santa Cruz Biotechnology, sc-245; WB and IF), rabbit anti-p-histone 3 (Santa Cruz Biotechnology, sc-8656-R; IF), mouse anti-CDK1-p34 (A17.1.1, Millipore, MAB8878; WB).

The following secondary antibodies from Jackson ImmunoResearch were used: Alexa Fluor® 488-conjugated goat anti-rabbit or anti-mouse IgG antibodies (115-547-003; FC and IF); Alexa Fluor® 594-conjugated goat anti-rabbit or anti-mouse IgG antibodies (115-517-003; IF). Other secondary antibodies used were: DyLightTM 800-conjugated goat anti-rabbit or anti-mouse IgG antibodies (ThermoFisher, WB) DyLightTM 488-conjugated goat anti-mouse IgG antibodies (35503, ThermoFisher; IF and FC) HRP conjugated goat anti-rabbit or anti-mouse IgG antibodies (Bio-Rad, WB).

### Flow-cytometry

Epithelial cells were harvested, fixed, and stained for DNA content (Propidium Iodide, PI) (25 μg/ml, 12 h), involucrin, K13, K16, and γH2AX as described [27]. For AOs flow-cytometry analysis, single cells were obtained as described [32], and fixed in 4% paraformaldehyde for 10 min (involucrin) or 70% ethanol (keratin K13) and stained as above. All experiments included parallel isotope control immunoglobulins (mouse IgGs (I-5381, Sigma-Aldrich; see Supplementary Fig. 9). After staining, all samples were firmly resuspended and filtered to minimise the presence of aggregates and then analysed on a CytoFLEX cytometer (Beckman Coulter). 10,000 events were gated in list mode and analysed for every sample.

### Comet assay

*Comet* assays or *single cell gel electrophoresis* were performed as described [21]. Cells were gently collected avoiding exposure to light. All incubations were performed in the dark at 4 °C. Briefly, 80000 cells were embedded in prewarmed (37 °C) 0.5% low melting point agarose (Invitrogen, 15517–014) and pipetted in slides (previously prepared and covered with one layer of 0.5% normal melting point agarose; Pronadisa, 8016). After solidification, another layer of 0.5% low melting point agarose was added to the slides. Samples were incubated for 1 h in cold lysis solution (10% DMSO, 1% Triton X-100) and 89% lysis buffer (pH 10, NaCl 2.5 M, EDTA 100 mM, Tris 10 mM, NaOH 200 mM). Then, electrophoresis was carried out at 25 V and 4 °C for 20 min. Finally, samples were washed in Tris 400 nM pH 7.5 and fixed in absolute ethanol. After DNA staining by RedSafe (1:500; InTron, 21141), samples were visualised under a fluorescent microscopy (Zeiss, Germany). DNA damage was quantitated by measuring the comet tail length in pixels by the ZEN 2012 programme (Zeiss).

### Immunofluorescence

For immunofluorescence, cells were cultured on glass coverslips, fixed with 3,8% formaldehyde in PBS for 10 min and permeabilised with −20 °C methanol for 5 min for involucrin staining or fixed and permeabilised with −20 °C cold methanol for 10 min. After two washes with PBS, cells were washed once with 0.5% Tween-20 PBS (5 min; P1379-250ML, Sigma-Aldrich). Then, cells were incubated 1 h in a wet chamber with primary antibody, washed as before and incubated for 1 h with secondary antibody in the dark. Cells were washed as before and incubated with 0.2 µg/ml DAPI (Sigma-Aldrich, D9542-1MG) 10 min. Finally, coverslips were mounted with ProLong Gold Antifade Reagent (Fisher, P10144) and visualised and photographed under Zeiss fluorescent microscopy.

For Supplementary Video 1, cells were grown on glass coverslips, fixed and stained as described above. Z-stack 3D digital images were reconstructed after frame collection by confocal microscopy (Nikon A1R, Melville, NY, USA; 20 × numerical aperture (NA) 0.75) and processed by NIS Elements software (AR, 3.2 64 bits; Nikon) as indicated in the legend of Supplementary Video 1.

For AOs immunofluorescence, organoids were collected, fixed, stained and mounted adapting the protocol described by Sachs et al. [32]. Briefly, AOs were removed from BME, fixed at RT for 2 h in 4% paraformaldehyde, permeabilised for 30 min 4 °C in 0.5% Triton X‐100 (Sigma) 2% BSA, and blocked for 15 min RT in 0.1% Tween-20, 2% BSA. Then, AOs were incubated overnight with primary antibodies at 4 °C, washed with PBS, and incubated for 1–2 h RT with secondary antibodies. Finally, AOs were washed again with PBS and milli-Q-purified water, mounted with ProLong Gold Antifade Reagent and visualised and photographed under Leica confocal microscopy, and processed using ImageJ.

### Quantitative PCR

Total RNA was isolated from two independent lung organoid cultures after a 24 h treatment with dimethyl sulfoxide (CT) or with Doxorubicin (DOXO) by using RNeasy kit (QIAGEN). Quantitative PCR (qPCR) was performed with Bio‐Rad systems in triplicate samples using SYBR green and the following primers: (K8 FW: GGAGAAGCTGAAGCTGGAGG; K8 RV: CCATGGACAGCACCACAGAT; GAPDH FW: CCAAAGTTGTCATGGATGAC; GAPDH RV: GTGAAGGTCGGTGTGAACGG). GAPDH was used as normalisation control.

### Western Blotting

Cellular pellets were incubated in lysis buffer (100 mM NaCl, 50 mM Tris pH 7,5, 10 mM EDTA, 1% NP40, 2.5 mM NaPPi, 5 mM NaF, 2.5 mM b-glycerophosphate, 1 mM DTT and supplemented with protease inhibitors from Boehringer Ingelheim, USA) for 10 min. Then, cells were mixed by pipetting and digested by 2 rounds of 5 min sonication. Cells were centrifuged at 15,000 × *g* for 15 min. The supernatant was collected as a soluble fraction and total protein quantification was carried out by a fluorometric system Qubit 4.0 (Life Technologies, Carlsbad, California, USA). An equal amount of protein (50 μg) was loaded onto the gel. Samples were separated by SDS-PAGE (12% acrylamide) and transferred to nitro-cellulose membranes. After blocking and incubating with primary (overnight, 4 °C) or secondary (2 h, room temperature) antibody, blots were finally subjected to enhanced chemiluminescence substrate (Cytiva Amersham™ ECL™ Prime Western Blotting Detection Reagent, RPN2232), following supplier’s protocol and visualised with FUSION Solo S-Western Blot & Chemi Imaging system (VILBER). See Original Western Blots in Supplementary Material.

### Statistical analyses

Data are mostly presented as mean ± SEM from at least two replicate samples (*n* = 2) and two or three independent experiments (*N* = 2–3) as indicated in each figure legend, unless stated otherwise. Sample size was chosen for qualitative significance and specificity as compared to control samples. Data sets were compared using an unpaired two-tailed Student’s t test. A *p*-value ≤ 0.05 was considered statistically significant. A higher *p*-value is indicated in figure legends when a clear tendency was observed. Damaged samples were excluded from analyses.

## Supplementary information

Supplementary Video 1

Supplementary Figures

Original Western Blots

## Data Availability

All data are available in the main text or in supplementary materials.
