# Supplementary material for: DNA damage triggers squamous metaplasia in human lung and mammary cells via mitotic checkpoints
Source: Cell Death Discov. 2023 Jan 21;9:21. doi: 10.1038/s41420-023-01330-3 (PMC9867756; doi:10.1038/s41420-023-01330-3)
Supplement: Supplementary file 2 — Supplementary Figures [file 41420_2023_1330_MOESM2_ESM.pdf]

Supplementary Information
San Juan et al

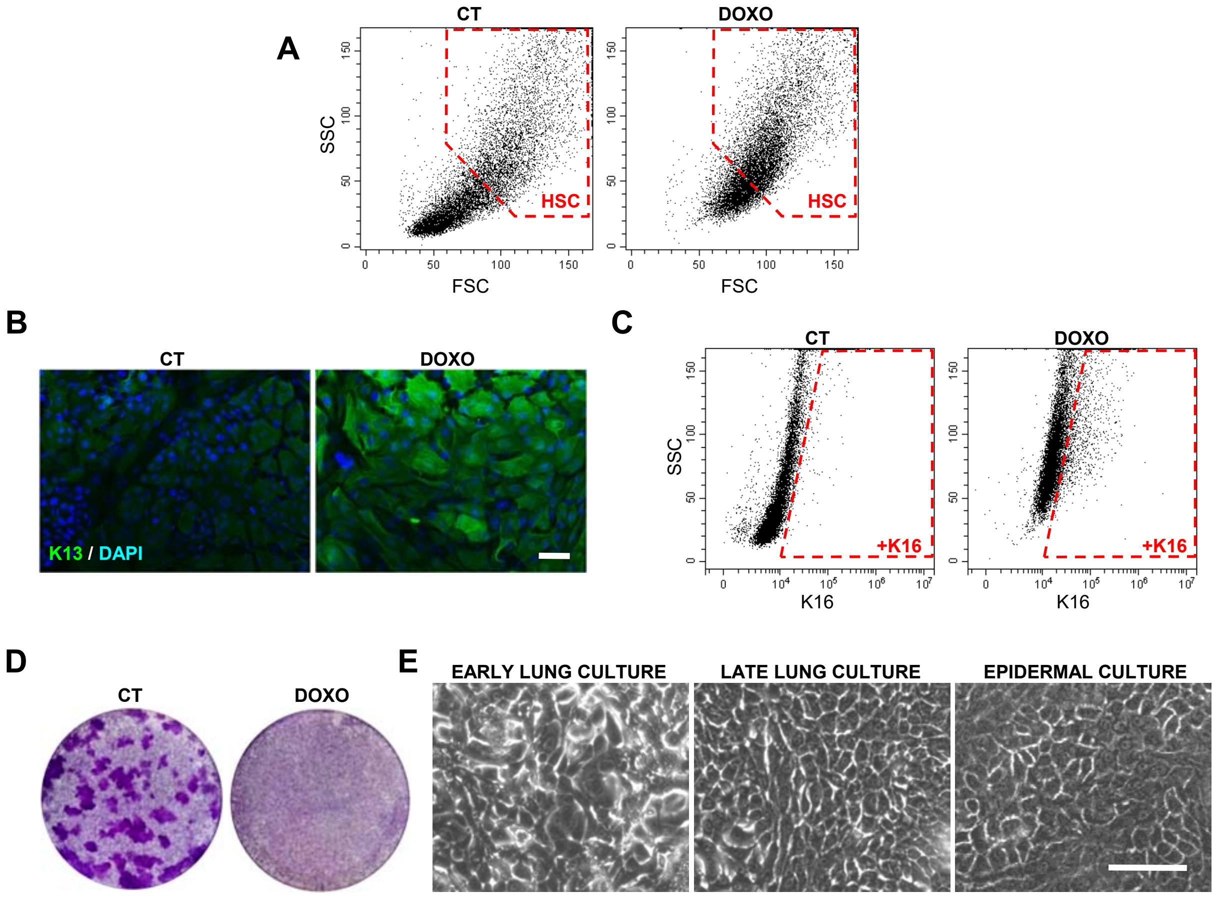

Suppl. Figure 1. Doxorubicin induces squamous metaplasia in human lung epithelial cells. Human primary lung epithelial cells were treated with dimethyl sulfoxide (CT) or with 0.5 µM Doxorubicin (DOXO) for 48 h as indicated. **A,** Representative flow-cytometry analyses for light scatter parameters (HSC, cells with high light scatter). **B,** Immunofluorescence for keratin K13 (green). Blue is nuclear DNA by DAPI. Scale bar, 50 µm. **C,** Representative flow-cytometry analyses for the squamous differentiation marker keratin K16 (+K16, positive cells). **D,** Clonogenic capacity of cells plated and drug-released after the indicated 48 h treatments. **E,** Phase contrast images of early (left) or late (center) cultures of primary human lung epithelial cells or primary human epidermal keratinocytes (right) in FAD-serum medium, hyperproliferative conditions. Scale bar, 50 µm. Positive cells by flow cytometry were gated according to negative isotype antibody control. Data are representative of 2-3 independent experiment from two different human individuals with similar results.

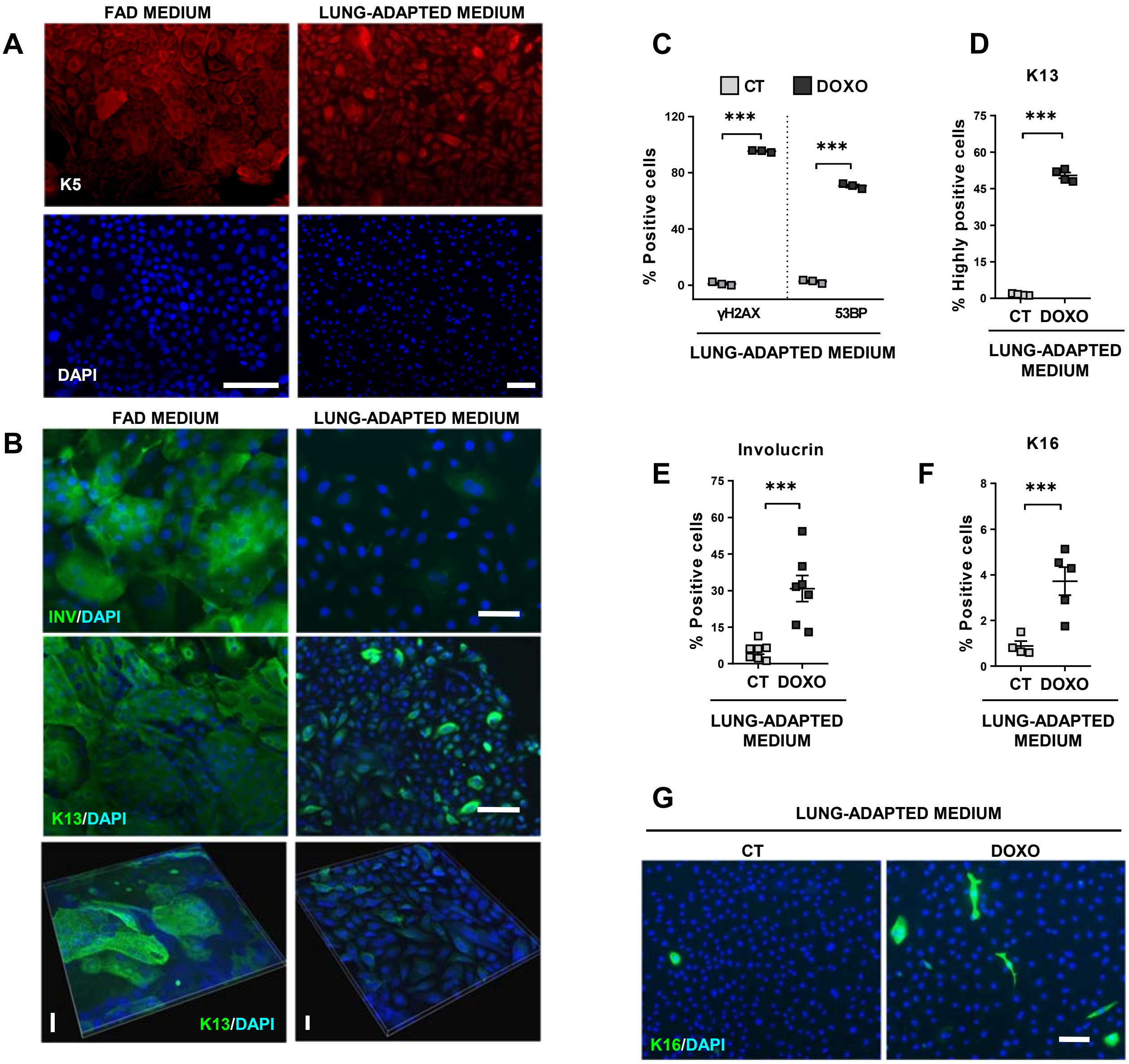

Suppl. Figure 2. Doxorubicin induces squamous differentiation in human lung epithelial cells in a lung-adapted serum-free medium. A-B, Human primary lung epithelial cells isolated and cultured in FAD (left) or in lung-adapted medium (righ). Immunofluorescence for K5 (A; red, top), involucrin (B; inv, green; top), or keratin K13 (B; green; center and bottom). Blue is nuclear DNA by DAPI. Scale bar, 100 µm (A), 50 µm (B, top, center). Bottom in B: Representative three-dimensional confocal microcopy reconstructions. Vertical scale bar in Z, 25 µm. **C-G,** Human primary lung epithelial cells in lung-adapted medium were treated for 48 h with dimethyl sulfoxide only (CT) or with 0.5 µM Doxorubicin (DOXO). (C-F) Quantitations by immunofluorescence for: (C) percent of γH2AX positive cells (left), percent of cells displaying a 53BP dotted pattern (right); (D) percent of keratin K13 positive cells; (E) percent of involucrin positive cells; (F) percent of keratin K16 positive cells. (G) Representative immunofluorescence of CT or DOXO cells for keratin K16 (green). Blue is nuclear DNA by DAPI. Scale bar, 100 µm. Data are mean ± SEM of 3-7 randomly selected fields, representative of 2 independent experiments. ***p < 0.001.

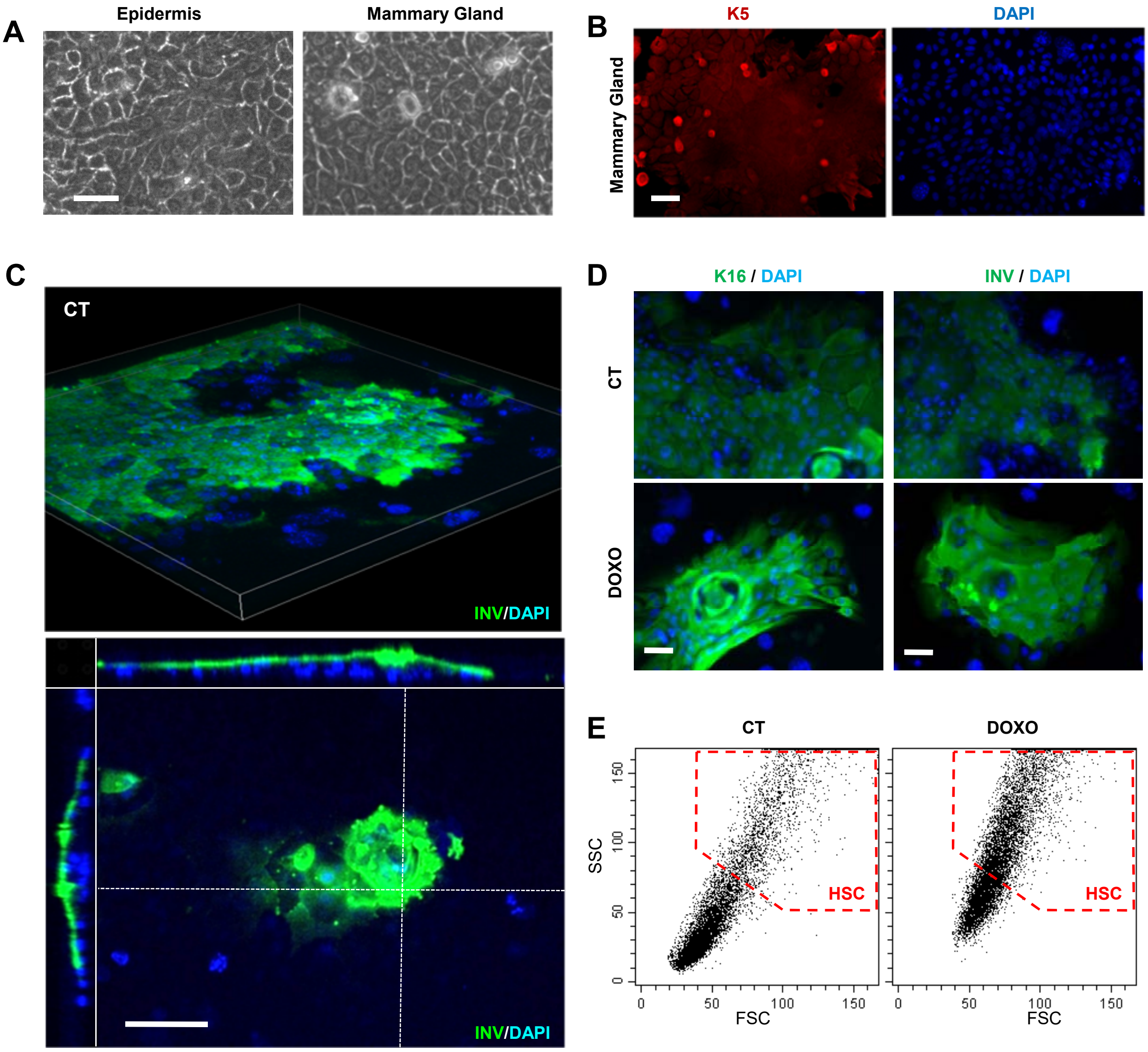

Suppl. Figure 3. Human mammary gland epithelial cells undergo squamous metaplasia upon hyperproliferative stimuli or DOXO treatment. A,B, Human epidermal keratinocytes or mammary gland epithelial cells, as indicated, cultured in FAD-serum medium. (A) Phase contrast microscopy images. (B) Immunofluorescence for K5 (red), or nuclear DNA by DAPI (blue). Scale bar, 50 µm (A,B). **C,** Top: Three-dimensional confocal microcopy reconstruction of immunofluorescence for involucrin (green) of untreated human mammary gland epithelial cells in FAD medium. Blue is nuclear DNA by DAPI. Bottom: orthogonal view of the same field (same colony in the top) by confocal analysis. **D-E,** Human primary mammary gland epithelial cells were treated with dimethyl sulfoxide (CT) or 0.5 µM Doxorubicin (DOXO) for 48 h as indicated. (D) Representative immunofluorescence for keratin K16 (green) or involucrin (green), as indicated. Blue is nuclear DNA by DAPI. Scale bar, 50 µm. (E) Representative flow-cytometry analyses for light scatter parameters of CT or DOXO-treated cells (HSC, cells with high light scatter). Data are representative of two independent experiment from two different human individuals with similar results.

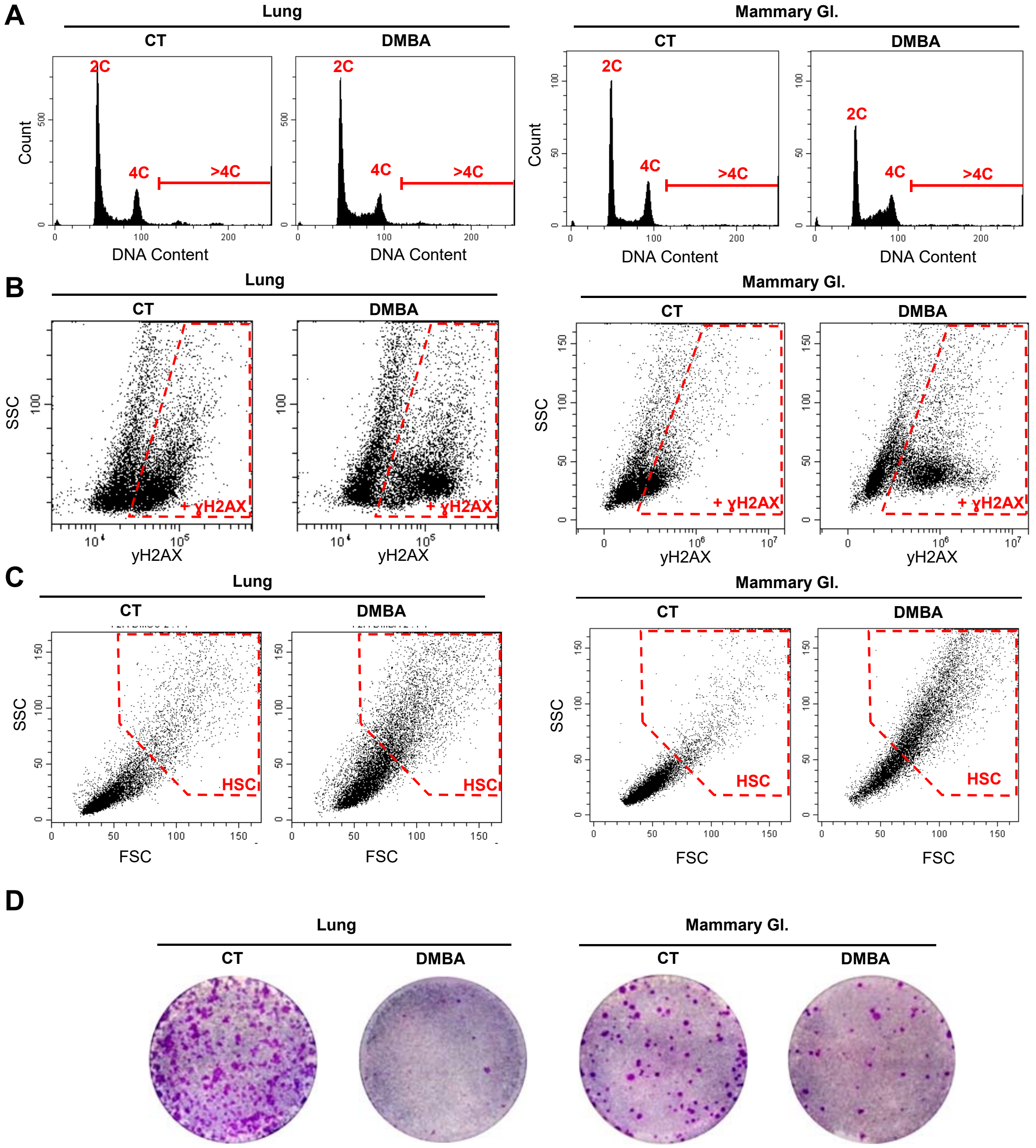

Suppl. Figure 4. Lung and mammary gland epithelial cells undergo squamous metaplasia in the presence of tobacco carcinogen DMBA. Human primary lung or mammary gland (mammary gl.) epithelial cells were treated with dimethyl sulfoxide (CT) or with 1 µgml^{-1} of 7,12-Dimethylbenz(a)anthracene (DMBA) for 24 h (A, B) or 72 h (C, D). **A,** Representative flow cytometry analyses of DNA content (2C, 4C and >4C indicate diploid, tetraploid and polyploid cells respectively). **B,** Representative flow-cytometry analyses for the DNA damage marker γH2AX (+γH2AX, positive cells). **C,** Representative flow-cytometry analyses for light scatter parameters of cells treated as indicated (HSC, cells with high light scatter). **D,** Clonogenic capacity of cells plated after after a 72 h treatment and drug-released. Positive cells by flow cytometry were gated according to negative isotype antibody control. Data are representative of 2-3 independent experiment from two different human individuals with similar results.

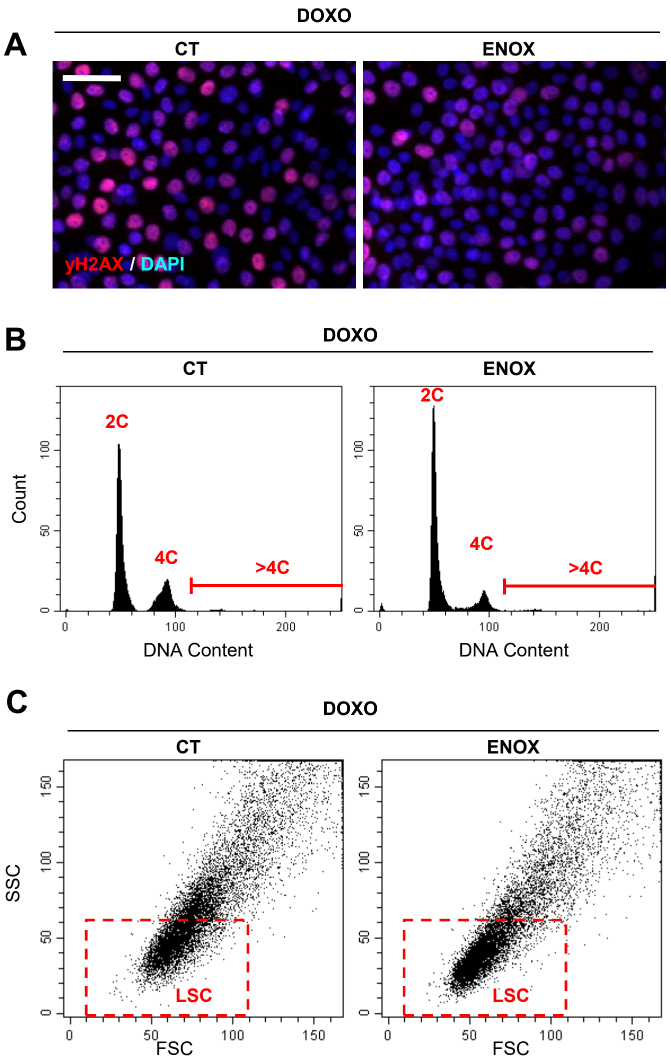

Suppl. Figure 5. Enhancement of DNA repair by ENOX suppresses Doxorubicin-induced squamous metaplasia in lung epithelial cells. Human primary lung epithelial cells were treated with 0.5 µM Doxorubicin (DOXO), dimethyl sulfoxide (CT) or 200 µM Enoxacin (ENOX). **A,** Immunofluorescence for γH2AX (red) of cells treated for 24 h as indicated. Blue is nuclear DNA by DAPI. Scale bar, 50 µm. **B,** Representative flow cytometry analyses of DNA content of cells treated for 24 h as indicated (2C, 4C and >4C indicate diploid, mitotic/tetraploid and polyploid cells, respectively). **C,** Representative flow cytometry analysis of light scatter parameters (LSC, cells with low light scatter) of cells treated for 24 h as indicated. Data are representative of 2-3 independent experiment from two different human individuals with similar results.

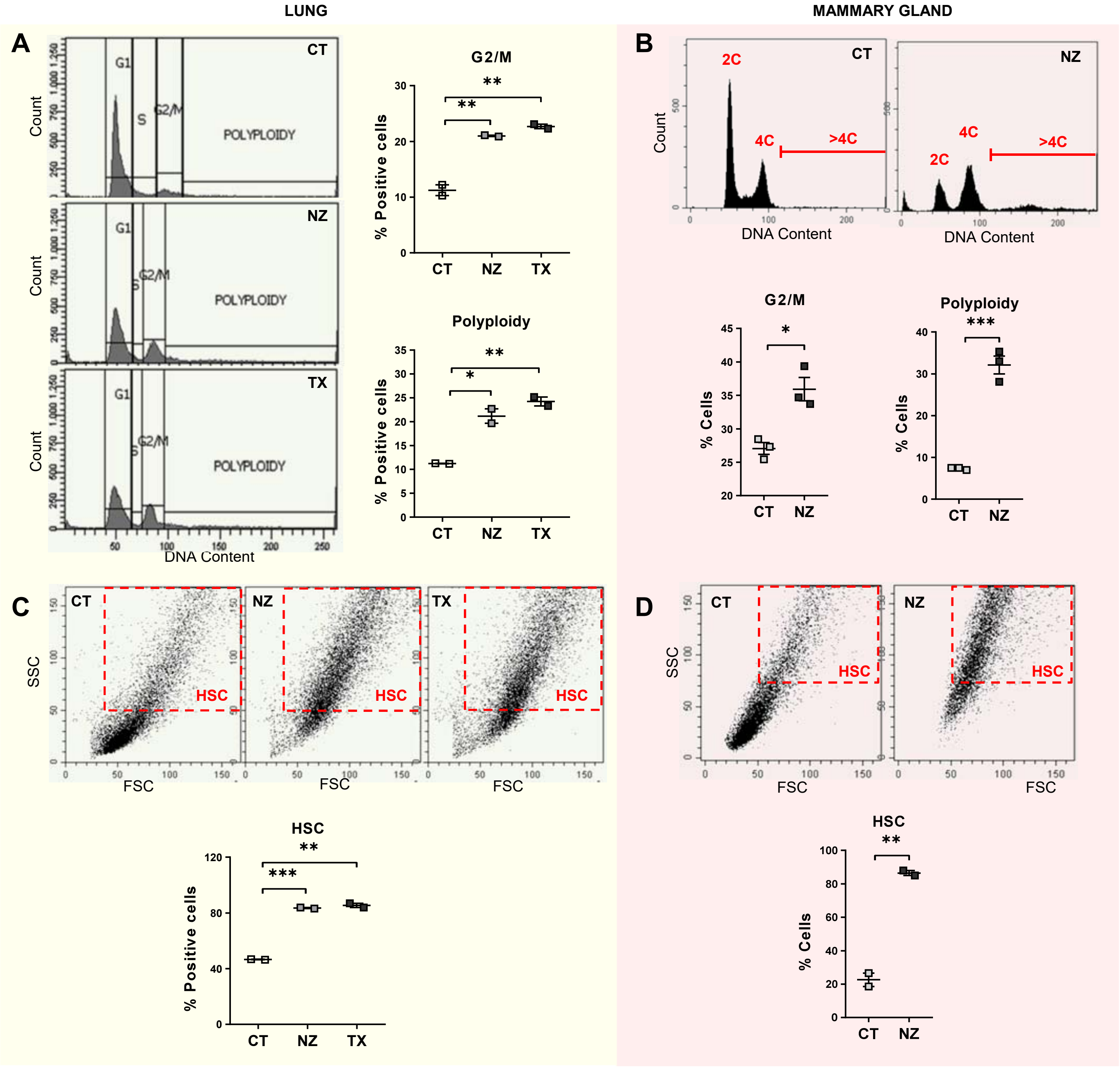

Suppl. Figure 6. Nocodazole or Taxol mitosis inhibitor induces polyploidisation and increased cell size and complexity in primary human lung and mammary gland epithelial cells. Human primary lung or mammary gland epithelial cells were treated with dimethyl sulfoxide only (CT), 20 µM Nocodazole or 200 µM Paclitaxel for 48 h. **A,** Left panels: Representative flow cytometry analyses of DNA content of lung epithelial cells treated as indicated. Right panels: scattered plots represent the percent of CT, NZ or TX cells in the G2/M phase of the cell cycle (top) or with >4C DNA content (bottom, polyploid). **B,** Top panels: representative flow-cytometry analyses of DNA content of mammary gland epithelial cells treated as indicated. Bottom panels: scattered plots represent the percent of CT or NZ cells in the G2/M phase of the cell cycle (left) or with >4C DNA content (right, polyploidy). **C,** Top panels: Representative flow-cytometry analysis for light scatter parameters of lung epithelial cells treated as indicated (HSC, cells with high light scatter). Bottom panel: percent of CT, NZ- or TX-treated cells with high light scatter. **D,** Top panels: representative flow cytometry analyses of light scatter parameters of mammary gland epithelial cells treated as indicated. Bottom panel: percent of CT or NZ cells with HSC. Data are mean ± SEM of 2-3 replicate samples, representative of 2 independent experiment from two different individuals. ***$p \leq 0.001$, **$p \leq 0.01$, *$p \leq 0.05$.

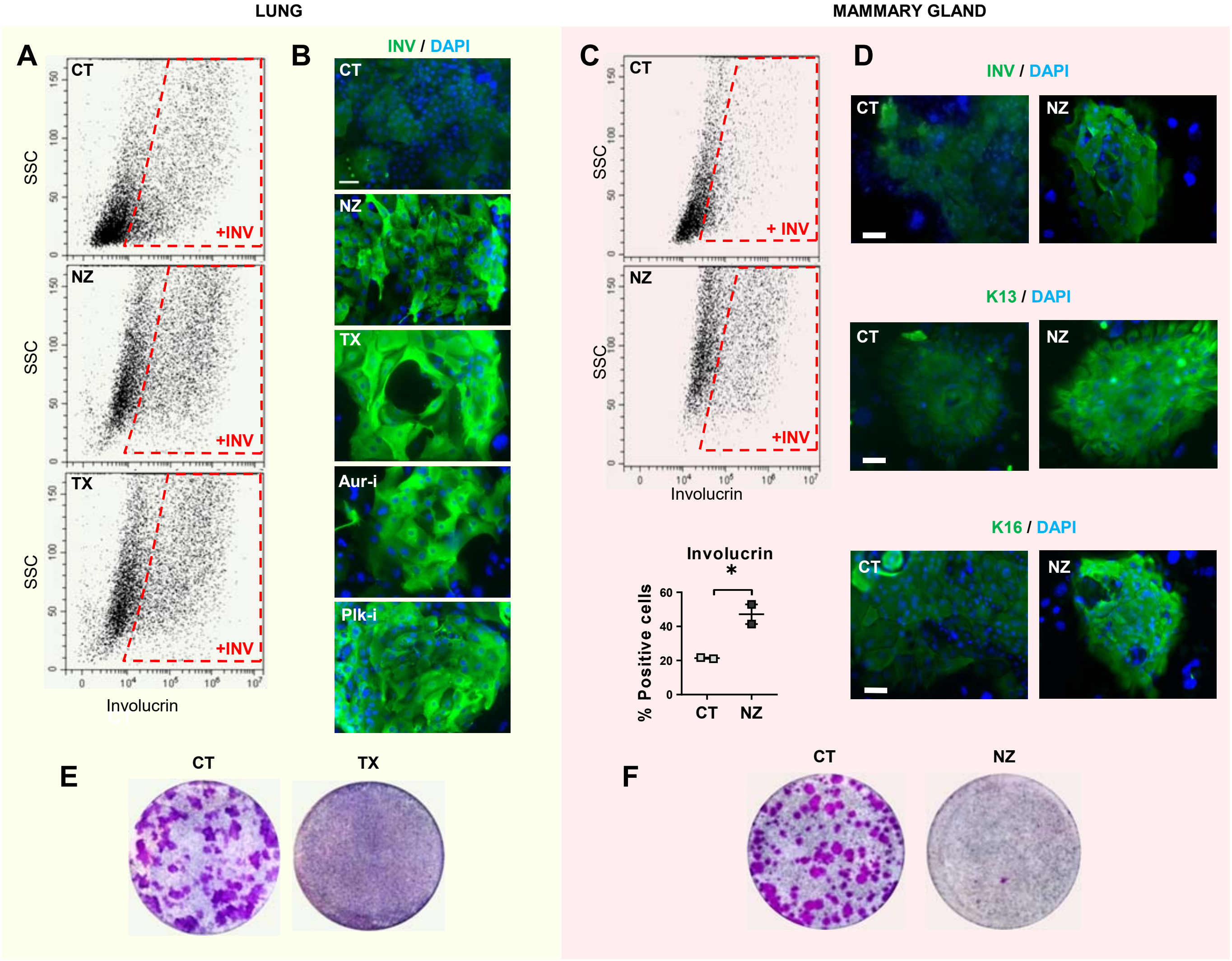

Suppl. figure 7. Mitosis inhibition induces squamous differentiation in lung and mammary gland epithelial cells. Human primary lung or mammary gland epithelial cells were treated as in Suppl. Figure 6, or with 2 µM ZM44739 (Aur-i, Aurora B kinase inhibitor), or 100 nM BI2536 (Plk-i, Polo-like kinase inhibitor) for 48 h as indicated. **A,** Representative flow cytometry analyses for the differentiation marker involucrin (+INV, positive cells) of lung epithelial cells treated as indicated. **B,** Immunofluorescence for involucrin (green) of lung epithelial cells. Blue is nuclear DNA by DAPI. Scale bar, 50 µm. **C,** Representative flow-cytometry analyses for the differentiation marker involucrin (+INV, positive cells) of mammary gland epithelial cells treated as indicated. Scattered plot displays the percentage of positive cells. **D,** Immunofluorescence for involucrin (INV, top), keratin K13 (center) or keratin K16 (bottom) of mammary gland epithelial cells treated as indicated. Blue is nuclear DNA by DAPI. Scale bar, 50 µm. **E,** Clonogenic capacity of lung epithelial cells plated and drug-released and after a 48 h treatment with CT or TX, as indicated. **F,** Clonogenic capacity of mammary gland epithelial cells drug-released and plated after a 48 h treatment with CT or NZ. Positive cells by flow cytometry were gated according to negative isotype antibody control. Data are mean ± SEM of duplicate samples, representative of 2 independent experiment from two different individuals. *p ≤ 0.05.

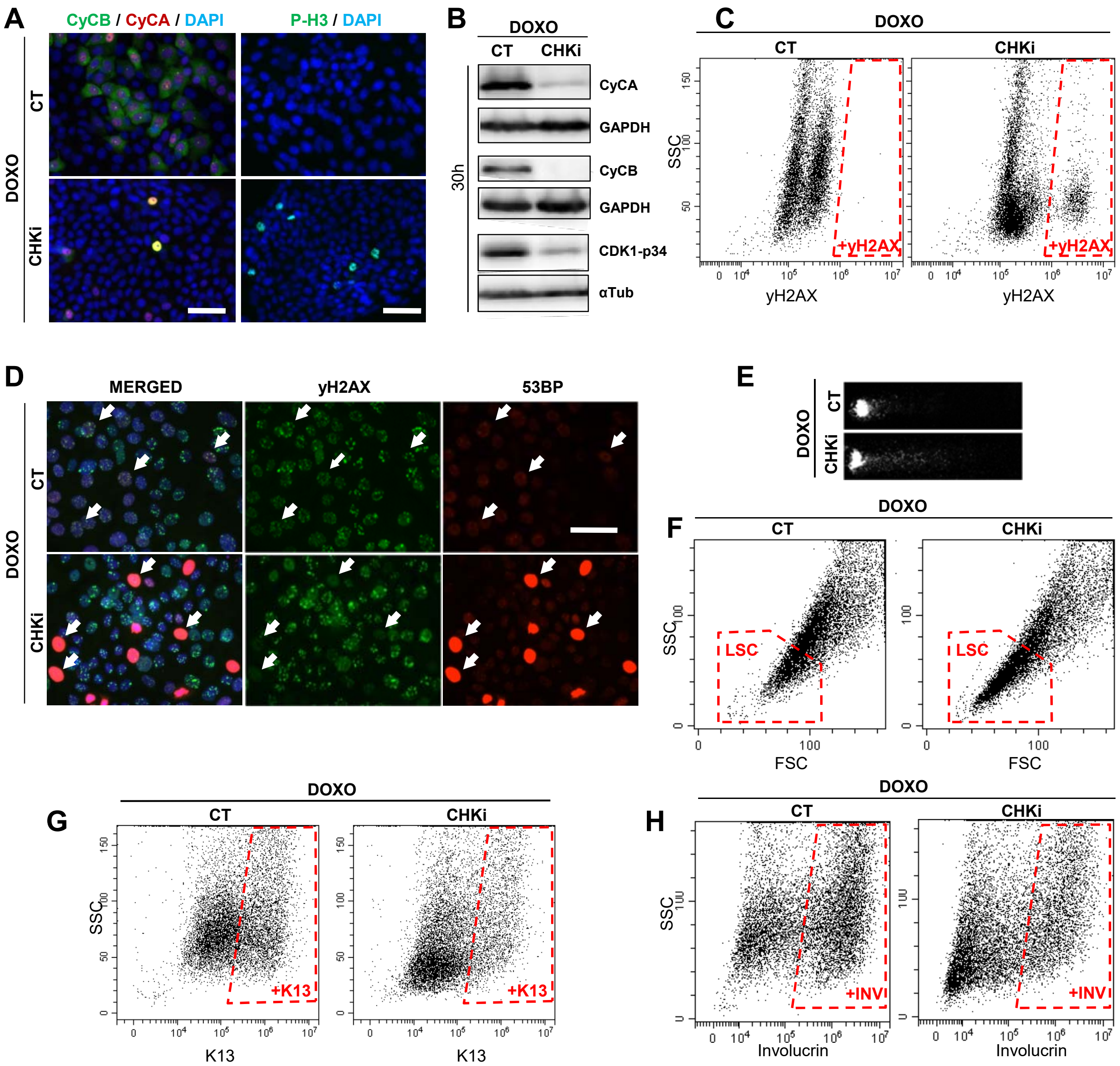

Suppl. figure 8. Chk1/Chk2 inhibition impairs DNA repair and attenuates Doxorubicin-induced squamous metaplasia in lung epithelial cells. Human primary lung epithelial cells were treated with 0.07 µM Doxorubicin (DOXO) or with dimethyl sulfoxide (CT) or 0.5 µM AZD7762 (CHKi, Chk1/Chk2 inhibitor). **A,** Left: Immunofluorescence for CyCB (green) and CyCA (red) of cells treated for 18 h as indicated. Right: Immunofluorescence for PH3 (green) of cells treated for 12 h as indicated. Blue is nuclear DNA by DAPI. Scale bars, 50 µm. **B,** CyCA, CyCB and Cdk1-p34 by Western blotting on cells treated for 30 h as indicated. Glyceraldehyde 3-phosphate dehydrogenase (GAPDH) and α-tubulin (αTub) are protein loading controls. **C,** Representative flow cytometry analyses for the DNA damage marker γH2AX of cells treated for 24 h as indicated (+γH2AX, highly positive cells). **D,** Double immunofluorescence for 53BP (green) and γH2AX (red) in cells treated for 24 h as indicated. Scale bar, 50 µm. Note (white arrows) that while γH2AX-positive CT cells show a dotted 53BP pattern typical of active DNA repair, strongly γH2AX-positive CHKi-treated cells were negative for 53BP. **E,** Representative images of DNA damage monitored by comet assays of cells treated for 24 h as indicated. **F,** Representative flow-cytometry analyses of light scatter parameters of cells treated for 48 h as indicated. LSC, cells with low light scatter. **G,** Representative flow cytometry analyses for the differentiation marker keratin K13 (+K13, highly positive cells induced by DOXO) of cells treated for 24 h as indicated. **H,** Representative flow cytometry analyses for the differentiation marker involucrin (+INV, positive cells) of cells treated for 48 h as indicated. Positive cells by flow cytometry were gated according to negative isotype antibody control. Data representative of 2-3 independent experiment from two different human individuals with similar results.

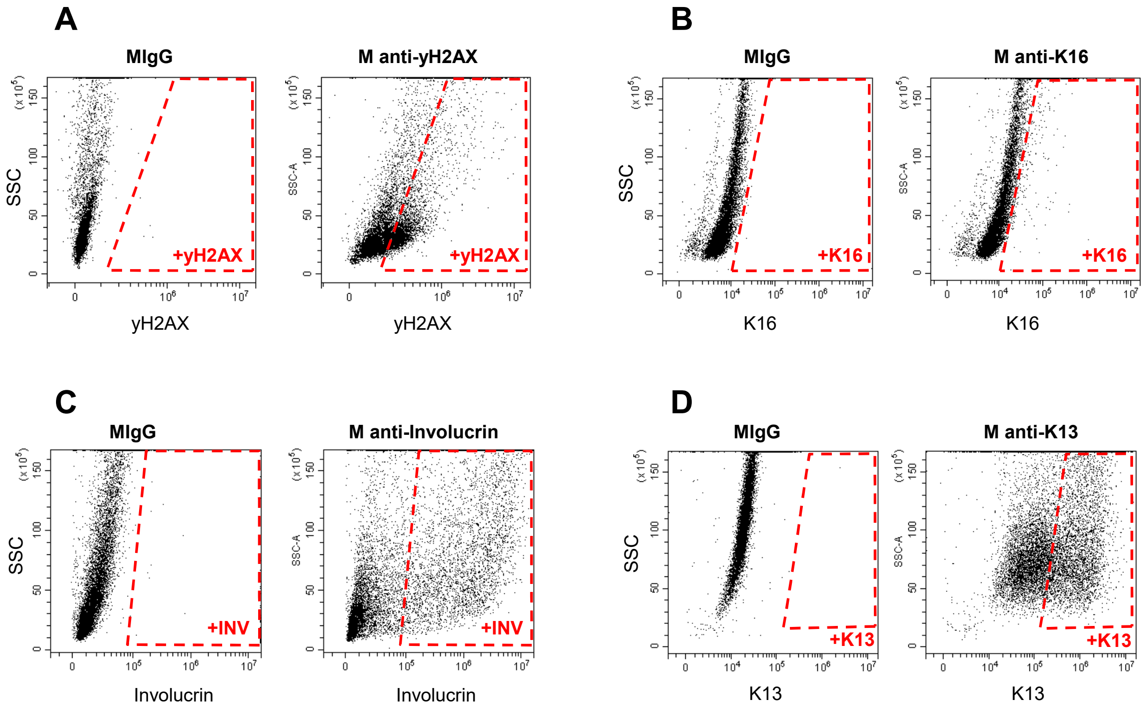

Suppl. Figure 9. Negative isotype control for antibodies used in flow cytometry analysis. Similar concentration of mouse (M) isotype negative immunoglobulins (IgG) was used as control for quantitation of positive cells in all antibody flow cytometry experiments. **A,** Mammary gland cells stained with MIgG or M anti-γH2AX antibody. **B,** Lung cells stained with MIgG or M anti-K16 antibody. **C,** Lung cells stained with MIgG or M anti-Involucrin antibody. **D,** Lung cells stained with MIgG or M anti-K13 antibody. Red broken line gates positive cells (A-C), or highly positive cells induced by DOXO (D).

Suppl. Video 1. Moving three-dimensional confocal reconstruction of mammary gland epithelial cells immunostained for involucrin (green). DAPI in blue for DNA. Frames were collected every 2 µm in the Z axis, which was scaled up 0.5 times (to 1.5 µm).
