# Supplementary figures and images for: DNA damage triggers squamous metaplasia in human lung and mammary cells via mitotic checkpoints
Source: Cell Death Discov. 2023 Jan 21;9:21. doi: 10.1038/s41420-023-01330-3 (PMC9867756; doi:10.1038/s41420-023-01330-3)

# Original western blots

SAN JUAN ET AL

# WB FIG. 6

SAN JUAN ET AL

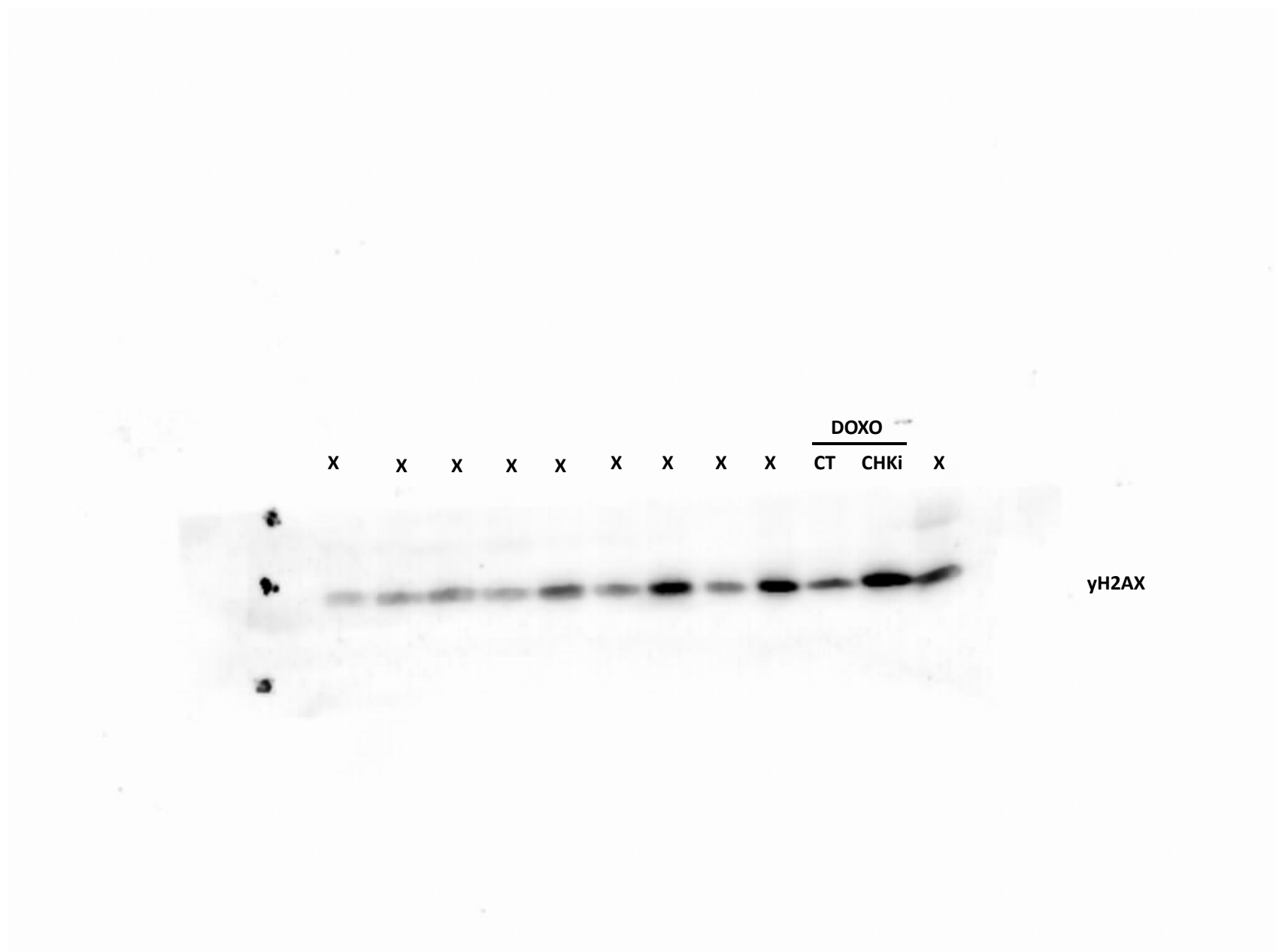

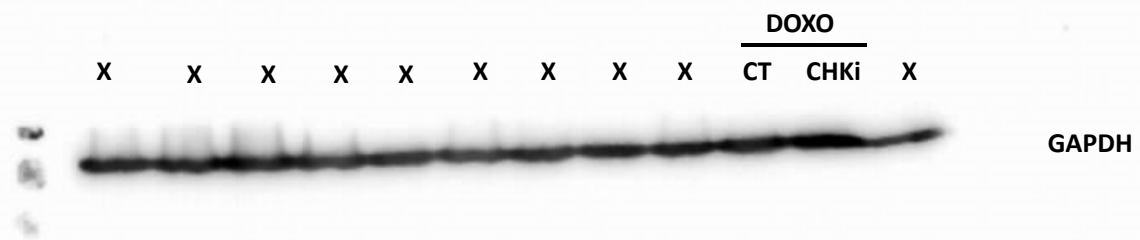

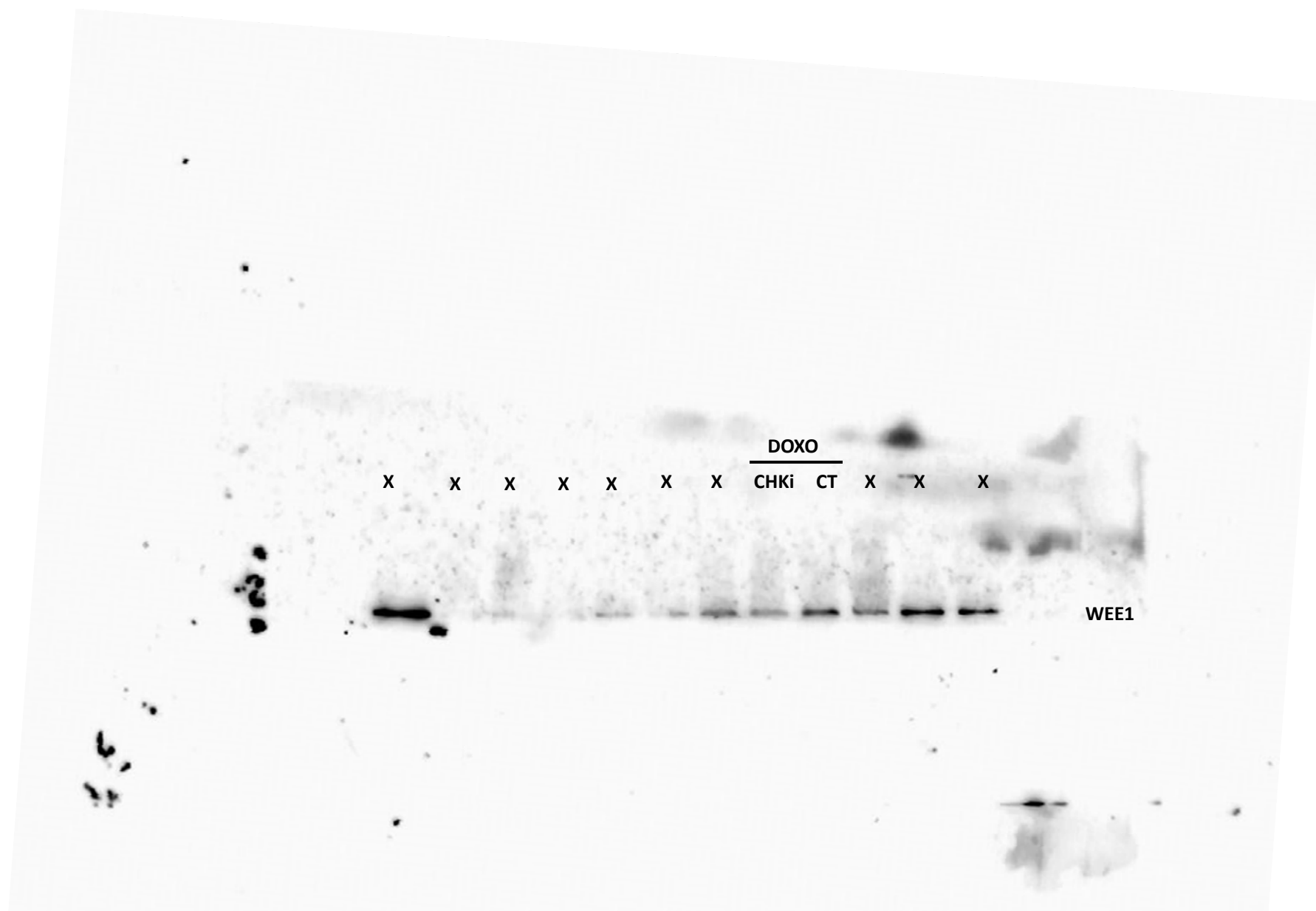

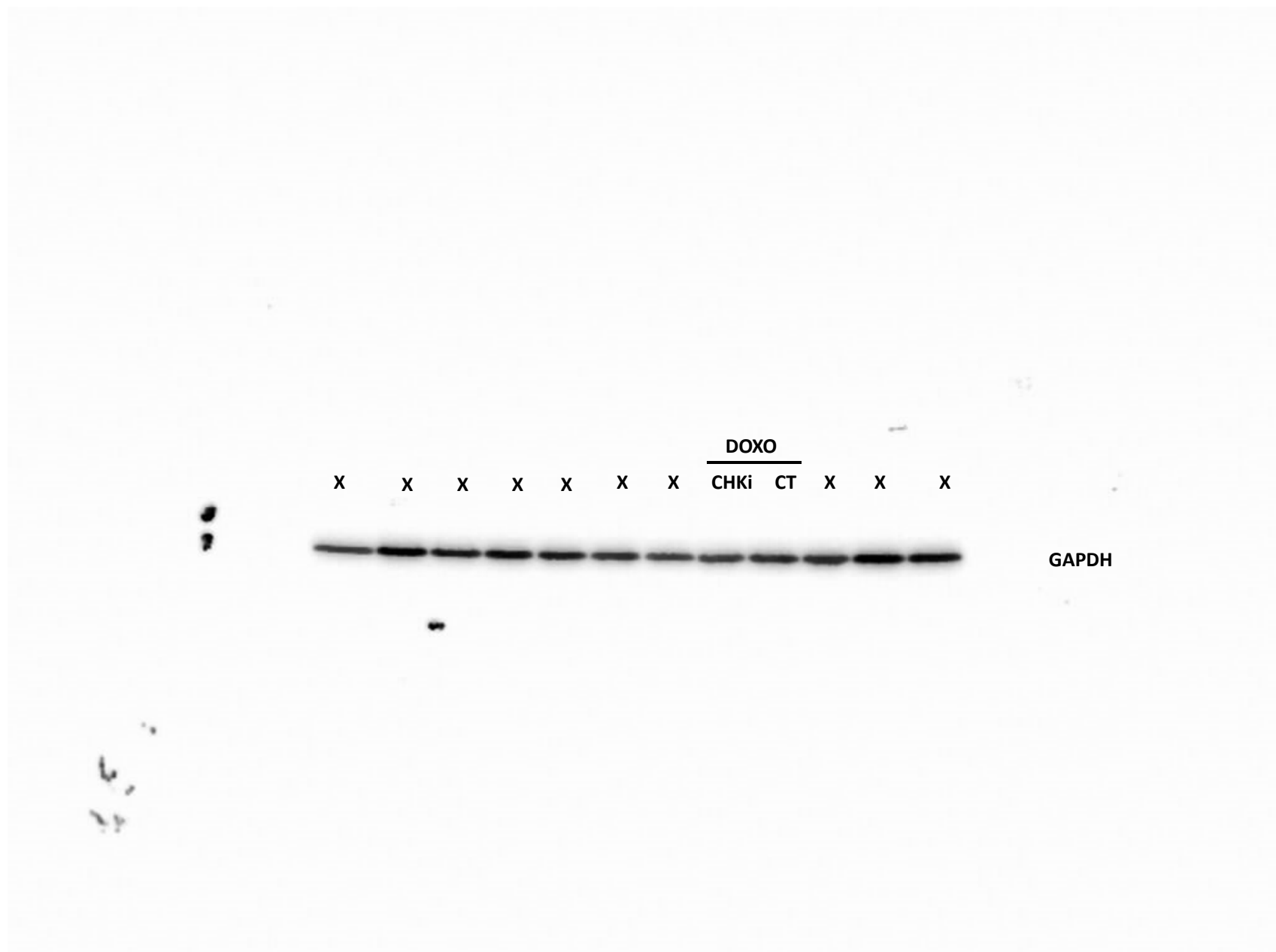

# WB SUPPL. FIG. 8

SAN JUAN ET AL

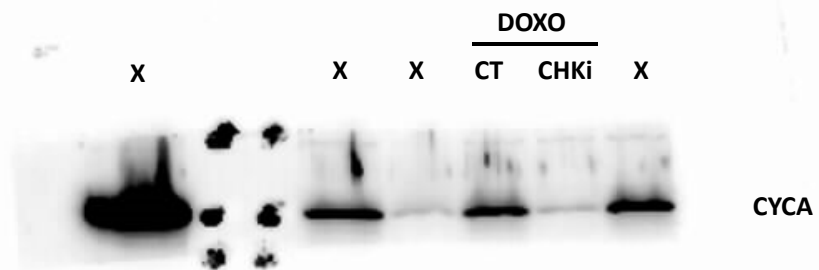

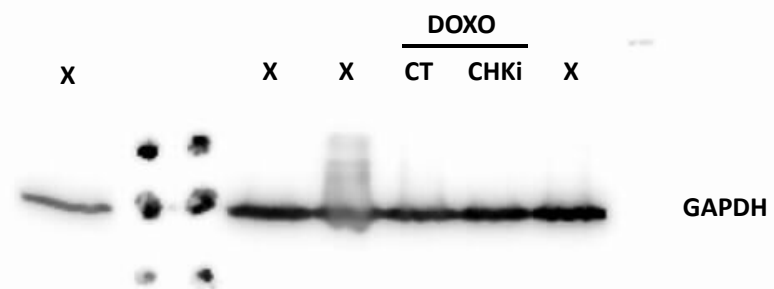

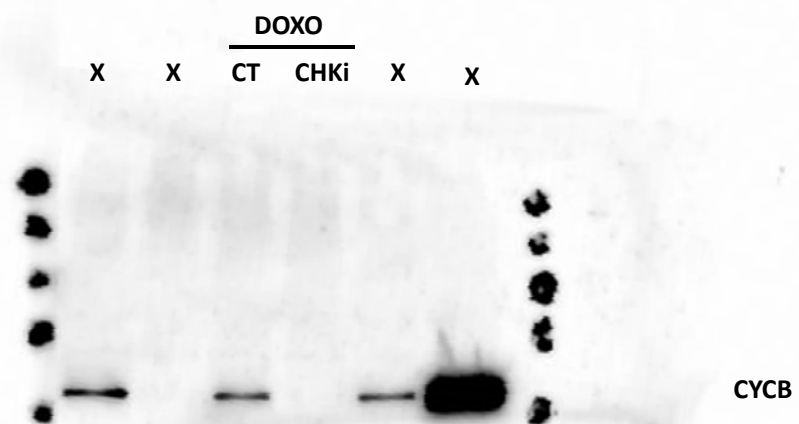

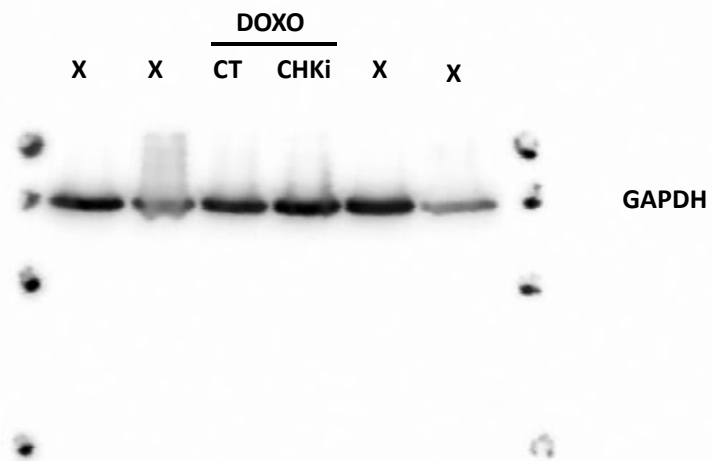

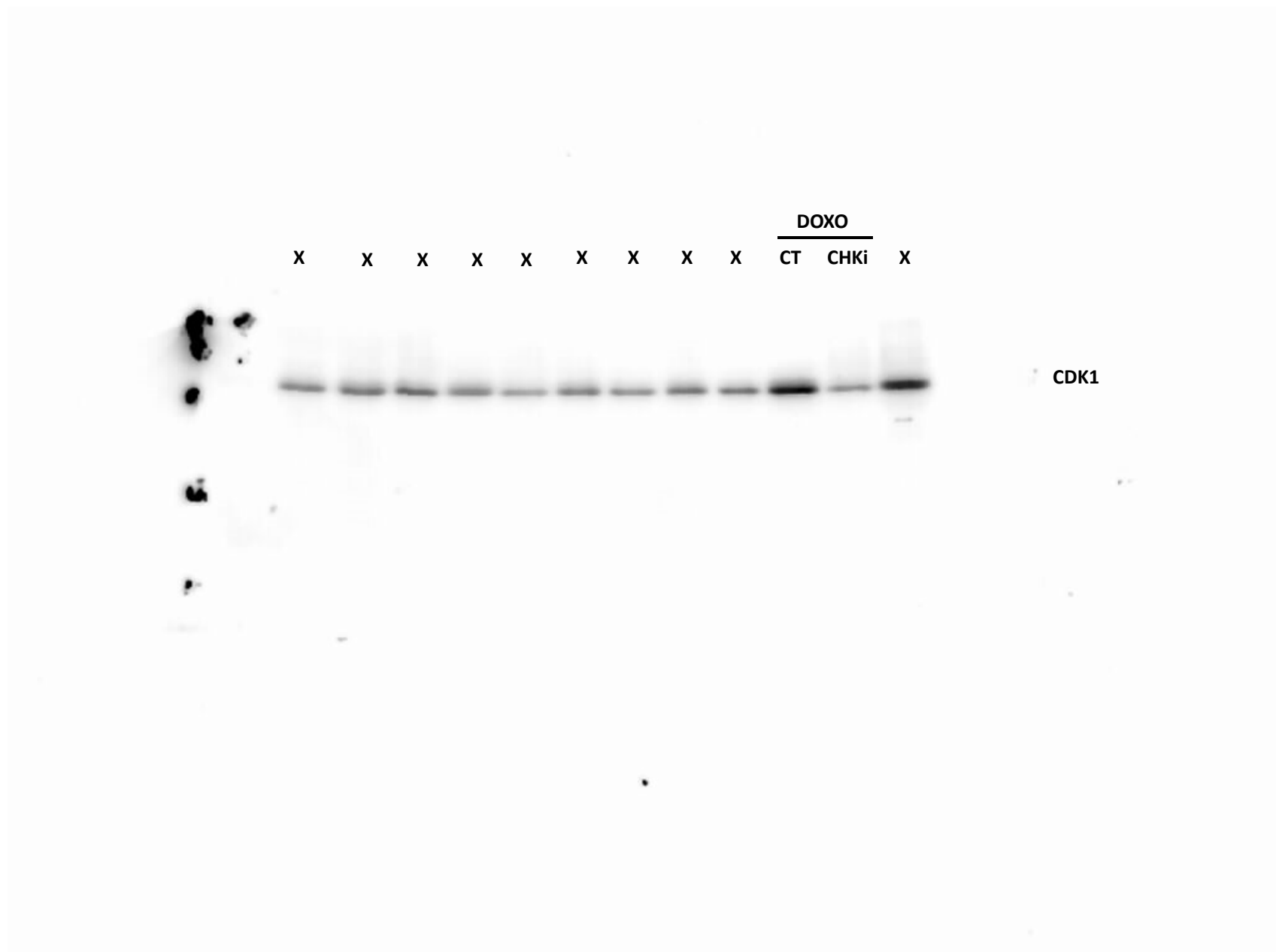

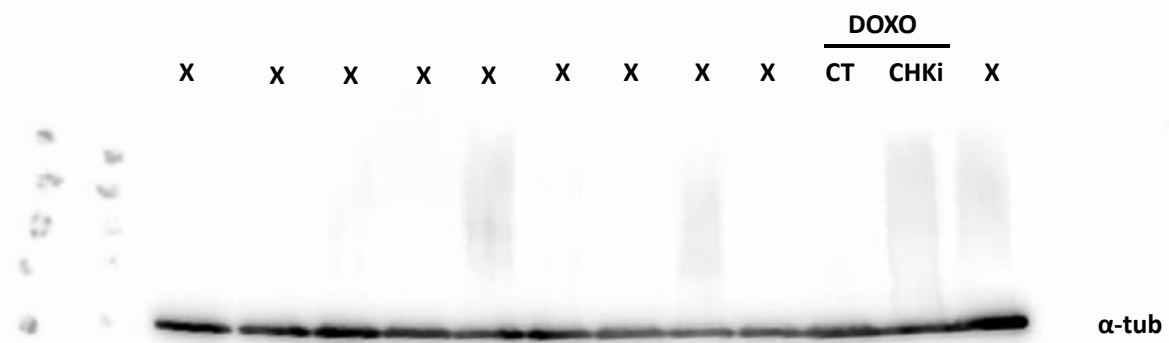

Supplement: Supplementary file 3 — Original Western Blots [file 41420_2023_1330_MOESM3_ESM.pdf]
